# Overcoming Resistance and Relapse in CAR-T and CAR-NK Cell Therapies: From Bench to Bedside

**DOI:** 10.34133/research.1068

**Published:** 2026-01-05

**Authors:** Yuhe Wang, Wenxia Shao, Jianhua Mao, Qing Ye

**Affiliations:** ^1^ Department of Nephrology, Children’s Hospital, and Liangzhu Laboratory, Zhejiang University School of Medicine, Hangzhou 310052, China.; ^2^ Affiliated Hangzhou First People’s Hospital, Westlake University School of Medicine, Hangzhou, China.; ^3^ Department of Nephrology, First Affiliated Hospital of Zhejiang Chinese Medical University (Zhejiang Provincial Hospital of Traditional Chinese Medicine), Hangzhou, Zhejiang 310003, China.; ^4^ Department of Laboratory Medicine, Children’s Hospital, and Liangzhu Laboratory, Zhejiang University School of Medicine, Hangzhou 310052, China.

## Abstract

As pioneering immunotherapy approaches, chimeric antigen receptor T cell (CAR-T) and natural killer cell (CAR-NK) therapies have shown notable clinical effectiveness when addressing different kinds of hematologic malignancies. For example, the application and efficacy of CAR-T cell therapy in acute lymphocytic leukemia, large B cell lymphoma, mantle cell lymphoma, and multiple myeloma have been widely recognized. In addition, the safety and feasibility of CAR-NK therapy when used to treat refractory/recurrent large B cell lymphoma have been verified. In particular, CD19-targeted CAR-T cell therapy has achieved marked efficacy and breakthrough progress in treating relapsed and refractory B cell leukemia and lymphoma. Although CAR-T cell therapy has achieved significant effectiveness in treating these diseases, patients still face challenges, including primary resistance and secondary recurrence after treatment. The complex mechanisms of resistance and recurrence involve multiple factors, such as target cells, CAR cell characteristics, and immune suppression conditions. This review examines resistance and recurrence mechanisms in CAR-T and CAR-NK therapies while exploring current therapeutic strategies and future research directions.

## Introduction

Chimeric antigen receptor (CAR) T cell therapy stands as a revolutionary cancer immunotherapy approach. Through genetic engineering techniques, T cells are modified to acquire the unique ability to precisely recognize and target specific antigens, thereby attacking target cells. When a CAR binds to a target antigen, it rapidly activates immune cells that carry the receptor, prompting them to release toxins to kill abnormal cells and secrete signaling molecules to recruit other immune components to participate in the response while supporting their own proliferation [1]. While the success of CAR-T cells in hematologic malignancies and solid tumors is well-established, their application is now expanding into areas such as autoimmune diseases (AIDs), where they enable the profound and sustained depletion of pathogenic B cells [2]. However, CAR-T cell therapy still faces challenges, including severe side effects, a long preparation cycle, high costs, and limited efficacy for solid tumors [3]. These limitations have spurred the development of alternative cellular platforms, most notably chimeric antigen receptor natural killer (CAR-NK) cells [4]. CAR-NK cells offer a number of advantages, including inherent antitumor activity, being unrestricted by the major histocompatibility complex (MHC), a lower risk of cytokine release syndrome (CRS) and immune effector cell-associated neurotoxicity syndrome (ICANS), a wide range of sources, and a certain degree of tolerance to the tumor microenvironment (TME) [5]. They also have the potential to be used to develop “off-the-shelf” therapies and may have broader application prospects in treating various diseases. Presently, multiple clinical investigations are underway. In a phase III study (NCT03391466) of ZUMA-7, the median follow-up was 47.2 months, and the event-free survival (EFS) and overall survival (OS) rates of the axicabtagene ciloleucel (axi-cel) group were significantly better than those of the standard salvage chemotherapy and transplantation groups [6]. Despite these advances, therapeutic durability remains a paramount concern. The increasing long-term follow-up data reveal a sobering reality: A substantial proportion of patients eventually relapse with either antigen-positive or antigen-negative disease. Consequently, understanding and overcoming the multifaceted mechanisms of resistance has become the central challenge in the field.

This article systematically summarizes the mechanisms and corresponding strategies of resistance to CAR-T and CAR-NK cell therapies, covering 3 key aspects: target cell factors, CAR-T cell factors, and immunosuppressive environment-related factors, aiming to chart a path toward more potent and durable cellular immunotherapies.

## Current Status of CAR-T and CAR-NK Cell Therapy

### Biological Basis of CAR-T/CAR-NK Cells

**Fig. 1.**
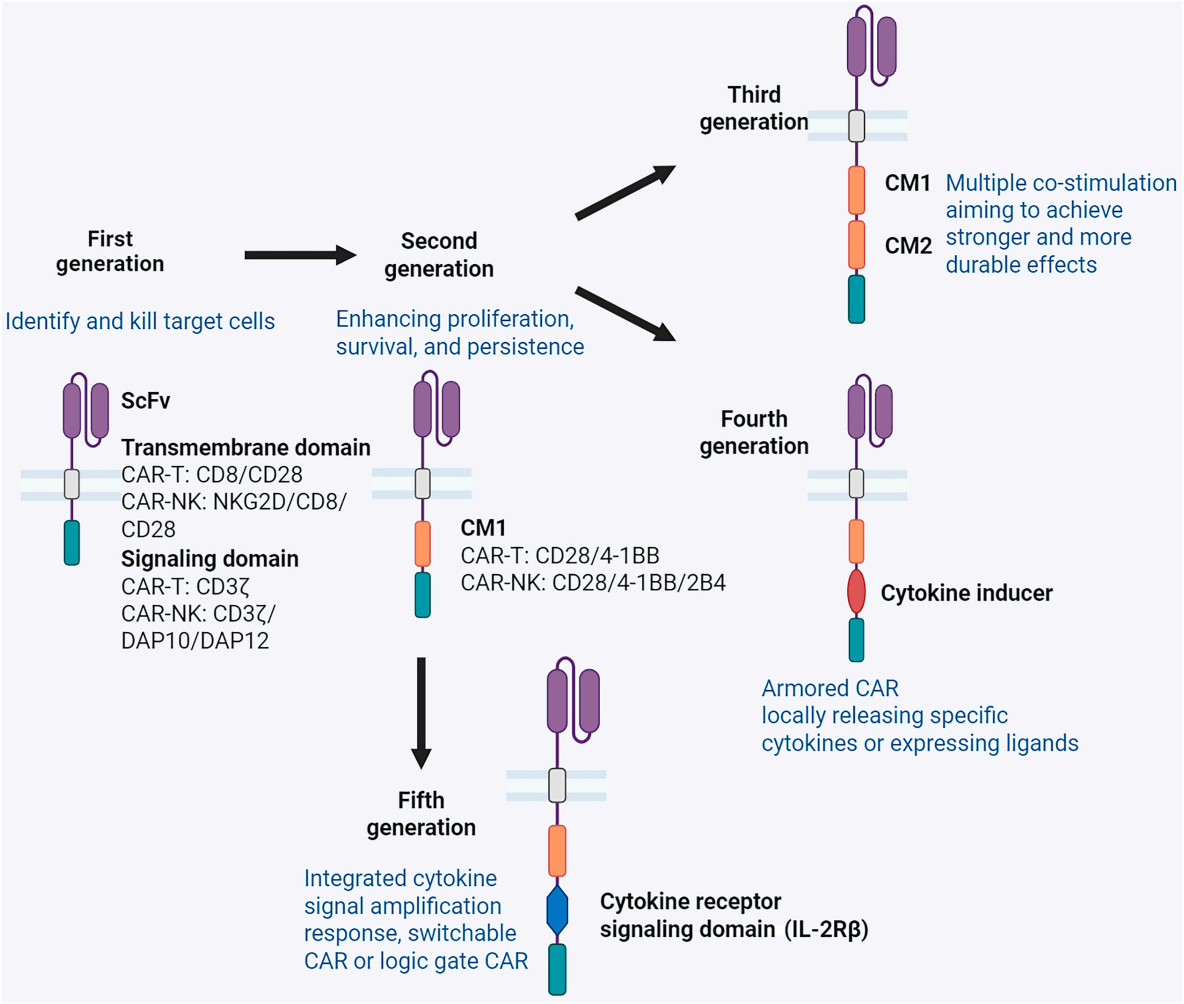
Five generations of the CAR structure. This diagram illustrates the structural progression of chimeric antigen receptors (CARs) across 5 generations, noting key components and their functional improvements. scFv, single-chain variable fragment; DAP10/DAP12, DNAX activation protein of 10/12 kDa (signaling adaptors in NK cells); NKG2D, natural killer group 2, member D (an activating receptor often used in CAR-NK designs); CM1/CM2, costimulatory module 1/2 (signifying different CMs). Created in https://BioRender.com.

The CAR molecule is an artificially designed transmembrane protein whose structure has undergone 5 generations of evolution since its initial proposal (Fig. 1). The current fifth-generation CAR structure comprises 5 key functional domains: the single-chain variable fragment (scFv), the transmembrane domain, a costimulatory molecule such as CD28 or 4-1BB, the CD3ζ signaling domain, and the cytokine receptor signaling domain [7]. This synthetic construct enables MHC-independent recognition and elimination of target cells, fundamentally distinguishing CAR-mediated killing from conventional T cell immunity.

When translated to NK cells, this CAR-mediated specific recognition synergizes with nonspecific recognition of the natural receptor system in NK cells, creating a dual-targeting system that bolsters both the efficiency and reliability of target cell killing [8,9]. Furthermore, the inherent biology of NK cells confers superior safety profiles for allogeneic applications. Since NK cells do not express a functional T cell receptor (TCR) and their activation remains MHC-independent, the risk of graft-versus-host disease (GvHD) is substantially lower. The ongoing refinement of CAR signaling cassettes thus remains a cornerstone in the development of more robust and predictable cellular products.

### Clinical data on drug resistance and relapse

The clinical success of CAR-T and CAR-NK therapies in hematological malignancies is invariably shadowed by the challenges of drug resistance and disease relapse, which manifest through 2 primary temporal and mechanistic patterns. Antigen-negative relapse refers to the recurrence of the disease state where target antigen expression has been lost or down-regulated. This type of recurrence is associated with changes in target cells and is more characteristic of late recurrence after CAR-T cell infusion. Antigen-positive relapse usually occurs early during the initial months following the successful establishment of remission and is usually linked to limited CAR-T cell persistence, indicating a lack of continuous CAR-T cell monitoring [10]. CAR-T and CAR-NK cell therapy has shown great therapeutic potential in various hematological tumors but is accompanied by drug resistance and recurrence (Table 1). CD19, the most widely used CAR-T cell target in B cell malignancies, has shown high remission rates in clinical trials. However, tumor cells often evade treatment through decreased or absent CD19 expression and subsequent relapse. Approximately 30% to 50% of patients develop resistance to CD19-targeted treatment within 12 months after CD19-targeted therapy, and this resistance pattern extends to other targets, including CD22 and BCMA [11]. CAR-NK cell therapy has demonstrated substantial efficacy in hematological malignancies, yet relapse and resistance persist as key clinical challenges that hinder long-term treatment success. For autoimmune diseases, the duration of drug-free remission following CAR-T cell therapy remains to be determined, and longer follow-up and clinical evidence are needed [12]. These observations collectively underscore that overcoming relapse requires a multi-pronged strategy targeting both the resilience of the target cell and the longevity of the therapeutic cell.

**Table 1.** Resistance and recurrence rates of CAR cell therapy in recent clinical trials

Clinical trial information	Disease	Target	Trial phase	Patients	Median follow-up time	CR	OS	PFS	Others	Ref.
NCT03090659 ChiCTRONH-17012285 CAR-T	MM	BCMA	I/II	74 After cyclophosphamide-based lymphodepletion therapy	65.4 months	54 (73%)	Median OS: 55.8 months 5-year OS: 49.1%	Median PFS: 18.0 months 5-year PFS rate: 21.0%	ORR:87.8% Remained relapse-free: 16.2%	[128]
NCT04555551 CAR-T	MM	GPRC5D	I	17	37 months	CR: 2 Response: 12 (71%)	Median OS: NR 3-year OS estimate: 59%	-	Median duration of response: 8.6 months GPRC5D loss: 6/10 (60%) patients at relapse	[129]
NCT04088864 NCT04088890 CAR-T	B-ALL	CD22	Ib	16 Refractory to 2 lines of therapy or to have relapsed after achieving prior CR	523 d	12 (75%)	Median OS: 523 d	Median PFS: 77 d	Ultimately relapsed: 9/12 (75%) CD22 down-regulation: 4/12 (33%)	[130]
NCT03896854 CAR-T	AML	CD19	II	10 Relapsed CD19-positive t (8;21) AML patients	64.6 months	10 (100%)	Median OS: 11.6 months 12-month OS rate: 45.0%	LFS: 3.8 months 12-month LFS rate: 46.7%	12-month CIR: 53.3% CD19-negative relapse: 5 CD19-positive relapse: 1	[131]
NCT04245722 CAR-NK	r/r B-ALL	CD19	I	86 Regimen A: FT596 monotherapy; *n* = 18 Regimen B: FT596 plus rituximab; *n* = 68	15.1 months	Regimen B: 25 (37%)	Regimen B median OS: 8.1 months	Regimen B median PFS: 3.5 months	Regimen B progressive disease: 23 (34%)	[132]
NCT05472558 CAR-NK	r/r LBCL	CD19	I	8 6 (75%) patients met the criteria for refractory aggressive LBCL	25 months	4 (50%) at day 30	Median OS: NR	Median PFS: 9.5 months	ORR at day 30: 62.5%	[115]

AML, acute myeloid leukemia; B-ALL, B cell acute lymphoblastic leukemia; BCMA, B cell maturation antigen; CR, complete response; CIR, cumulative incidence of relapse; FT596, an induced pluripotent stem cell (iPSC)-derived CAR NK cell therapy; GPRC5D, G protein-coupled receptor class C group 5 member D; LBCL, large B cell lymphoma; LFS, leukemia-free survival; MM, multiple myeloma; NR, not reached; ORR, overall response rate; OS, overall survival; PFS, progression-free survival

## Mechanism of Resistance and Recurrence

### Target cell factors

#### Antigenic escape and target cell heterogeneity

**Fig. 2.**
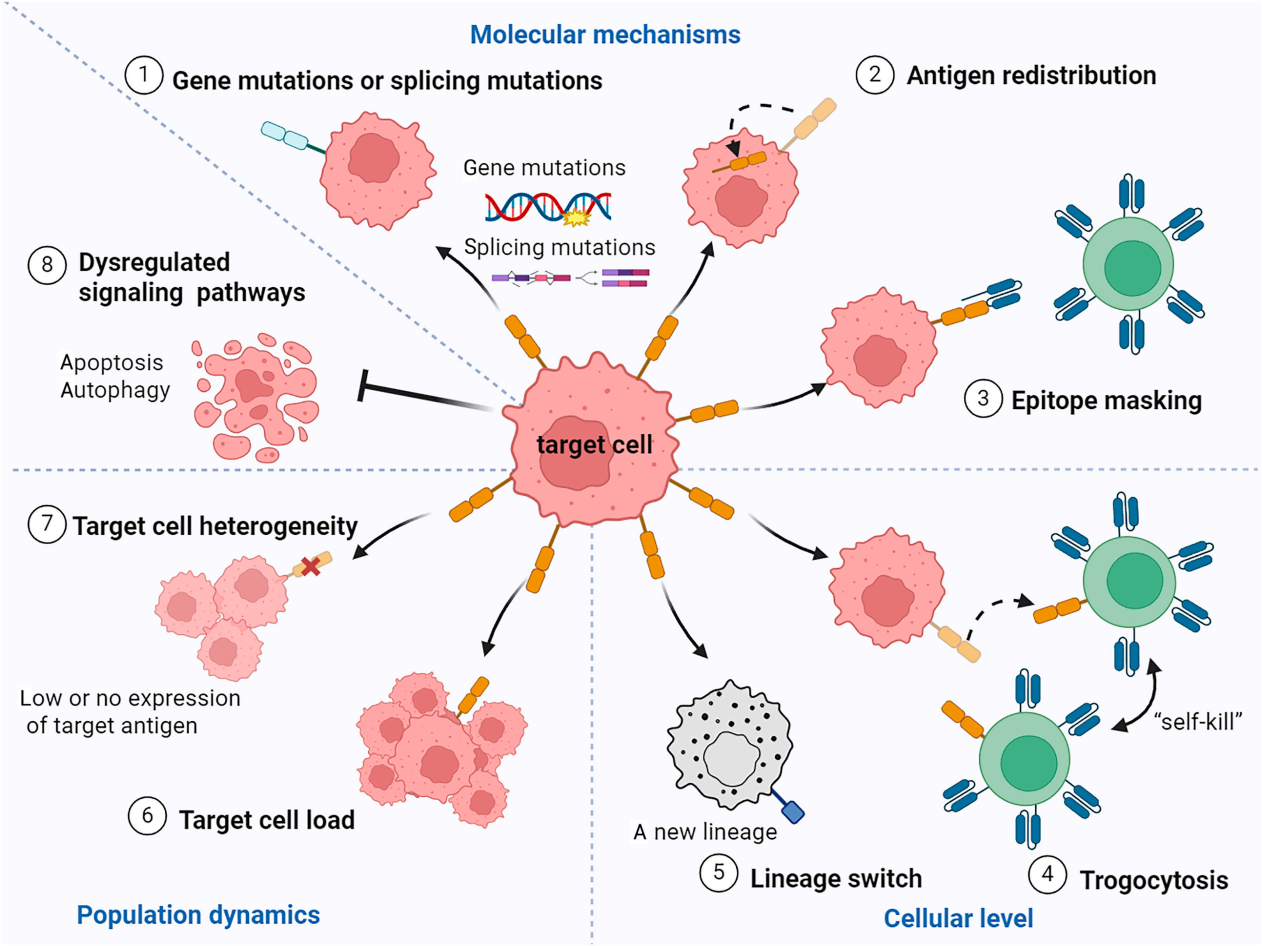
Target cell factors influencing resistance and recurrence mechanisms. This diagram outlines 7 mechanisms of antigenic escape, a process through which target cells evade immune detection. (1) Gene mutations or splicing mutations: Changes in the genetic code or mRNA processing that impact the target antigen. (2) Antigen redistribution: The internalization or shedding of the target antigen from the cell surface. (3) Epitope masking: The physical obstruction of the antigenic epitope by other molecules. (4) Trogocytosis: Trogocytosis involves the transfer of the target antigen from the tumor cell to the immune cell, making both the tumor cell undetectable and potentially causing immune cell fratricide. (5) Lineage switch: A shift in the cell’s differentiation state results in the loss of the original lineage-specific target antigen. (6) Target cell load: The quantity or density of target antigens, which can affect therapeutic efficacy. (7) Variability in antigen expression, including low or absent expression among a population of target cells. (8) Dysregulated signaling pathways: Activation of pro-survival/anti-apoptotic signals (e.g., BCL-2 and MCL-1) and autophagy confer resistance to immune cell-induced death. Created in https://BioRender.com.

Target cell factors are considered the most common mechanisms for disease recurrence. Tumor cells down-regulate or entirely eliminate the surface presentation of target antigens through various mechanisms, including antigen gene mutation, alternative splicing, epitope masking, trogocytosis, and lineage switching, thereby preventing CAR-T cells from recognizing and attacking them (Fig. 2) [13]. Here, we delve into a comprehensive analysis of pertinent factors, ranging from molecular mechanisms and cellular interactions to population dynamics and functional adaptations.

At the molecular level, sustained immunologic pressure from single-target therapies drives multiple antigen escape mechanisms, which is essentially a manifestation of co-evolution between CAR cells and target cells under Darwinian selection pressure. In CD19-targeting CAR-T cell therapy for B cell acute lymphocytic leukemia (B-ALL), persistent single-target immunotherapy may induce selection pressure that triggers CD19 gene mutations, leading to reduced or absent surface expression [14,15]. The inherent genomic instability and alternative splicing mechanism of tumor cells increase the frequency of antigen mutations, which in turn leads to the down-regulation of antigens and the appearance of neoantigens, hindering CAR-T cell recognition. Beyond genetic alterations, antigen redistribution, referring to the process of antigen transfer from the cell membrane surface to a different subcellular location, has been confirmed as a potential mechanism for reducing target antigen expression [13]. In addition, tumor cells achieve epitope masking by down-regulating or losing CD19 antigen expression, thereby protecting themselves from CAR-T cell recognition and attack. Ruella et al. [16] documented this epitope masking phenomenon in B-ALL patients receiving CD19 CAR-T cell therapy. CAR genes are transferred accidentally to leukemia cells during manufacturing and express proteins that block CD19 epitopes, leading to immune escape and drug resistance.

For cellular interactions, trogocytosis involves membrane fragmentation and bidirectional molecular interaction exchange between CAR-T cells and target cells, leading to reversible antigen loss and antigen transfer to CAR-T cells [17]. This membrane transfer mechanism induces self-elimination in CAR-T cells, reducing the activity and function of T cells [18]. Recent researches have revealed that through trogocytosis tumor cells acquire CAR molecules from CAR-T cells, resulting in CAR depletion, subsequent CAR-T cell dysfunction, short-term antigen loss, and epitope masking [19]. These phenomena may similarly interfere with autoimmune diseases [17]. While less characterized in CAR-NK biology, trogocytosis likely represents a conserved resistance mechanism across cellular immunotherapy platforms.

At the cellular level, tumors demonstrate remarkable phenotypic plasticity through lineage switching, which refers to the transformation of tumor cells from their original lineage to a new lineage under intense immunological selection pressure. The most common transformation is from B-ALL to acute myeloid leukemia (AML). Persistent CD19 CAR-T cell immune pressure can induce lineage switching, leading to drug resistance [20]. In addition, a retrospective study revealed that 17 patients (23%) with B-ALL transferred to mixed phenotypic acute leukemia (MPAL)/acute leukemias of ambiguous lineage (ALAL) and highlighted that lineage switching rapidly emerges after immunotherapy [21]. Such transformations underscore the limitations of therapies targeting differentiation antigens without addressing underlying oncogenic drivers.

Owing to genetic variations, environmental differences, and reversible changes in cellular characteristics, target cells exhibit phenotypic and functional heterogeneity, particularly in solid tumors [22]. This provides an “evolutionary reserve” for tumor cells to resist CAR-T therapy under Darwinian selection pressure. Among these target cells, there may be preexisting clones with low or no target antigen expression, which are initially in the minority. However, under the continuous selection pressure of CAR cells, antigen-positive dominant clones are effectively eliminated, while these antigen-negative minor clones are rapidly amplified and become the main population of residual tumors, ultimately leading to disease recurrence [23]. This reflects co-evolution between target and CAR cells: Therapeutic cells reshape tumor populations via selection, while tumors adapt through clonal evolution to form a dynamic confrontation. In AID therapy, CD19 CAR-T cells mainly target B cells but have a limited effect on long-lived plasma cells (LLPCs) with negative or low expression of CD19, potentially leading to disease relapse [24]. Therefore, single-target CAR-T cells cannot recognize and attack all target cells. Excessive load can lead to the inability to clear all target cells, but in autoimmune diseases, the B cell load is significantly reduced compared to tumor patients, leading to the swift clearance of antigen-positive cells [12]. Collectively, these mechanisms highlight that overcoming target cell-mediated resistance requires addressing both the dynamic adaptability of individual cells and the complex architecture of cell populations.

#### Dysregulated signaling and apoptotic pathways

Beyond antigenic escape, tumor cells develop resistance by evading the core cytotoxic mechanisms of CAR-T cells, which execute killing primarily through the perforin/granzyme pathway, death receptor signaling [e.g., FAS, tumor necrosis factor receptor 1 (TNF-R1), and TRAIL and/or its death receptors (TRAIL-R1/2), and cytokine-mediated pathways like interferon-γ (IFN-γ) [25]. A pivotal resistance mechanism is the constitutive activation of intrinsic pro-survival and anti-apoptotic signaling within tumor cells. The up-regulation of proteins such as BCL-2 and MCL-1 elevates the threshold for mitochondrial apoptosis, rendering tumor cells resistant to CAR-T cell-induced death. This is clinically significant, as high MCL-1 expression in multiple myeloma (MM) has been shown to directly limit the efficacy of anti-BCMA CAR T cells [26]. Concurrently, impaired death receptor signaling can cripple this extrinsic apoptotic pathway, leading to progressive CAR-T cell dysfunction [25].

Emerging evidence highlights the role of cellular homeostasis pathways like autophagy in facilitating evasion. In B cell leukemia and lymphoma models, pharmacological inhibition of autophagy or knockout of the key gene RB1CC1 significantly sensitized tumor cells to CD19 CAR-T cell killing [27]. This study revealed that cancer-intrinsic autophagy mediates resistance by suppressing TNF-α–TNF-R1-mediated apoptosis and modulating STAT1/IRF1-induced chemokine signaling. Other critical pathways further contribute to this resilient phenotype. Activation of nuclear factor κB (NF-κB) and phosphatidylinositol 3-kinase (PI3K) signaling promotes cell survival and resistance to apoptosis, while stabilization of β-catenin helps maintain a stem-like state associated with therapy resistance and tumorigenicity. Collectively, these interconnected signaling networks, spanning apoptosis regulation, metabolic adaptation, and stemness maintenance, form a robust shield against immune attack. Therefore, co-targeting these fundamental oncogenic pathways represents a promising strategy to resensitize refractory tumors to CAR-T cell therapy.

### CAR-T factors

**Fig. 3.**
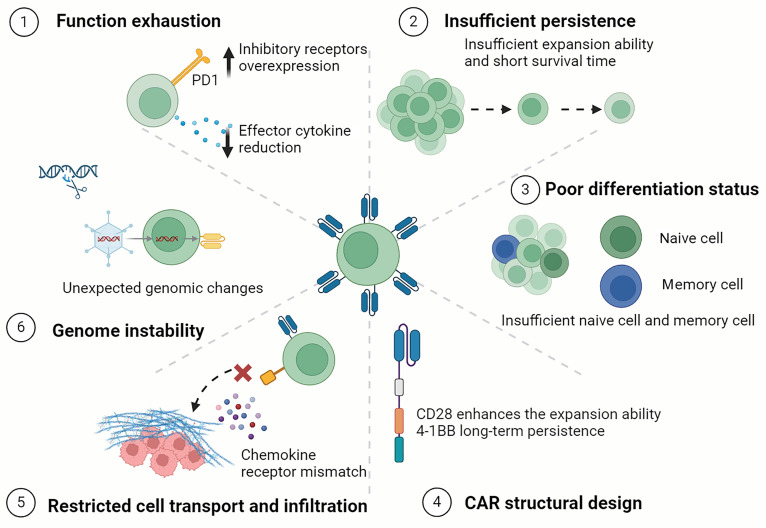
CAR cell factors contributing to resistance and recurrence mechanisms. This schematic outline 6 factors contributing to CAR-T cell dysfunction or failure. (1) T cell exhaustion manifests through elevated expression of multiple inhibitory receptors of inhibitory receptors (e.g., PD-1, TIM-3, and LAG-3) and reduced secretion of effector cytokines (e.g., IFN-γ and TNF-α). (2) Insufficient persistence is due to the inability of CAR-T cells to survive long-term in vivo, resulting from poor expansion capabilities and a short lifespan. (3) A poor differentiation status is characterized by an unfavorable composition of CAR-T cell subsets at infusion, particularly a low proportion of naive and memory cells, which are essential for long-term persistence. (4) The design of the CAR molecule itself (e.g., the selection of CMs such as CD28 or 4-1BB) significantly affects function. CD28 domains increase rapid expansion, whereas 4-1BB domains promote long-term persistence. (5) The inability of CAR-T cells to migrate to and penetrate tumor sites can be caused by a chemokine receptor mismatch between CAR-T cells and the microenvironment. (6) Genome instability refers to unexpected genomic alterations (e.g., mutations and insertional oncogenesis) in engineered CAR-T cells, which may impact their safety and function. CAR, chimeric antigen receptor; PD-1, programmed cell death protein 1; TIM-3, T cell immunoglobulin and mucin-domain containing-3; LAG-3, lymphocyte-activation gene 3; IFN-γ, interferon-γ; TNF-α, tumor necrosis factor-α. Created in https://BioRender.com.

Limitations rooted in the CAR-T cells are associated with the function, persistence, and adaptability of CAR-T cells and often trigger early antigen-positive relapse (Fig. 3). Certain aspects of vector design may impact gene transfer efficiency, which may also affect functional capability of the ultimately manufactured CAR-T cell product.

#### Function exhaustion

A central barrier to durable efficacy is T cell exhaustion, a hypofunctional state precipitated by chronic antigen exposure [28]. Under persistent antigen stimulation, T cells display reduced proliferation capacity, diminished effector functions, and the overexpression of various inhibitory receptors. Inhibitory receptors, such as programmed cell death protein-1 (PD-1), mucin domain-containing protein-3 (TIM-3), and lymphocyte-activated gene-3 (LAG-3), are up-regulated, and the secretion of effector cytokines (such as IFN-γ, TNF-α, and IL-2) is decreased [29].

Continuous antigen stimulation causes epigenetic reprogramming of CAR-T cells, leading to an exhausted state that is largely irreversible, even after antigen removal [30]. Beyond persistent antigen exposure, the development of exhaustion in both endogenous and CAR T cells is coordinated through chronic inflammatory signals and microenvironmental stressors. Factors including hypoxia-inducible factor-1α (HIF-1α), vascular endothelial growth factor (VEGF), and transforming growth factor-β (TGF-β) synergistically contribute to the establishment of this dysfunctional state, further hindering treatment effectiveness [31].

This convergence of signals establishes exhaustion not merely as a transient dysfunction but as a fixed cellular fate that severely impairs long-term disease control, necessitating strategies that simultaneously target multiple signaling pathways to achieve durable reversal.

#### Insufficient persistence and poor differentiation status

The transient lifespan of CAR-T cells in vivo, marked by a rapid expansion phase followed by a precipitous decline, fundamentally limits their ability to prevent relapse [32]. Patient-derived T cells, especially after multiple therapies, exhibit shorter telomeres and limited proliferative potential, making them more prone to terminal differentiation and resulting in insufficient persistence in the body. Additionally, research has shown that that CAR-T cell loss is attributed to CD8^+^ T cell immunity against CAR transgenes, while the antigenicity of mouse-derived scFvs may reduce CAR-T cell persistence [33]. The persistence and memory phenotype of CAR-T cells prove critical for the immune response. The abundance of early-lineage cells, such as naive T cells and stem memory T cells, is critical for the proliferation ability and long-term efficacy of CAR-T cells. Additionally, different phenotypic subpopulations of CD4^+^ and CD8^+^ T cells exhibit varying proliferative potentials and lifespans, and a balanced CD4/CD8 CAR T cell ratio of 1:1 can enhance antitumor activity [34]. Clinical studies have shown that exhausted CD4^+^ CAR-T cells are correlated with early disease recurrence and express cytotoxic and exhaustion markers [35]. Thus, the initial differentiation state and cellular composition of the product are key determinants of its in vivo destiny.

#### Restricted cell transport and infiltration

While CAR-T cells readily access circulating tumor cells in hematologic malignancies, their efficacy in solid tumors is critically hampered by failed delivery. In solid tumors where abnormal blood vessel networks and extracellular matrix (ECM) barriers exist, infused CAR-T cells fail to effectively migrate and infiltrate tumor sites, thereby preventing their capacity to recognize and eliminate cancer cells. In addition, mismatched chemokine–chemokine receptor pairs provide another mechanistic explanation for the insufficient recruitment of CAR-T cells into tumors [36]. Consequently, even potent CAR-T cells remain ineffective if they cannot physically localize to the tumor site.

#### CAR structural design and genome instability

The differentiation, metabolism, and persistence of T cells are profoundly influenced by structural design, particularly the selection of costimulatory domains (CMs). Preclinical research indicates that the CD28 CM usually endows T cells with preferential glycolytic metabolism and enhances the expansion ability of CAR-T cells, whereas the 4-1BB CM tends to maintain mitochondrial adaptability and promote memory cell differentiation and long-term persistence [37,38]. Vector insertion mutations are not only problems leading to drug resistance and relapse but also safety issues. Genome engineering strategies utilizing CRISPR/Cas9 for CAR cell production may introduce unexpected and irreversible genomic changes in the product cells [39]. Therefore, the pursuit of next-generation CARs must balance optimized signaling with ensuring genomic fidelity.

### Factors related to the immunosuppressive environment

**Fig. 4.**
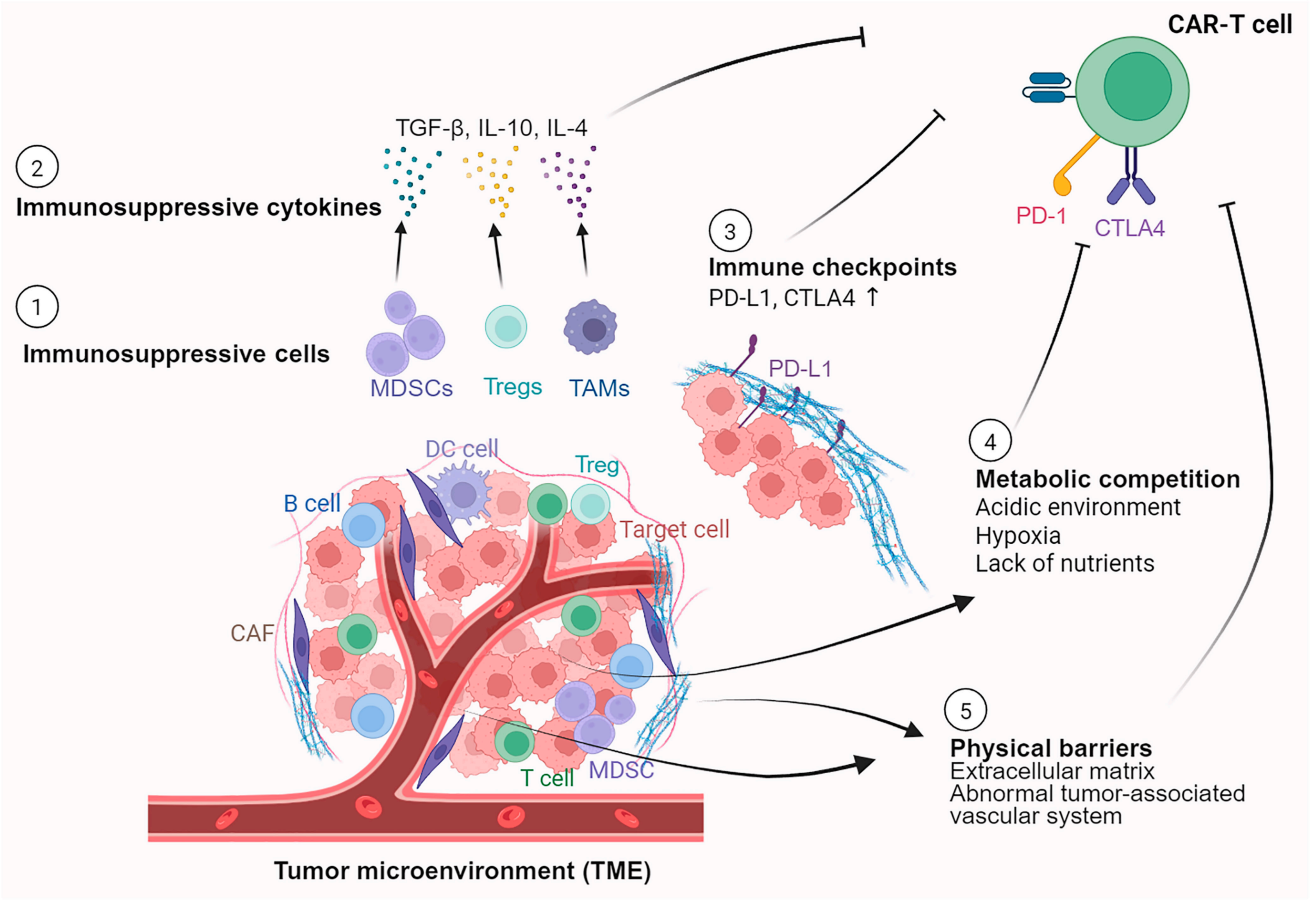
Factors contributing to the immunosuppressive environment include mechanisms of resistance and recurrence. This schematic highlights the major immunosuppressive factors within the tumor microenvironment (TME). These findings illustrate the presence of immunosuppressive cells, such as myeloid-derived suppressor cells (MDSCs), regulatory T cells (Tregs), and tumor-associated macrophages (TAMs), which secrete immunosuppressive cytokines such as TGF-β, IL-10, and IL-4. Compared to CAR-T cells, CAR-NK cells exhibit heightened sensitivity to TGF-β-mediated inhibition, and their function is markedly regulated by IL-10 secreted by MDSCs. A close interaction between a dendritic cell (DC), a Treg, and a target cell is depicted, emphasizing cell-mediated suppression. The engagement of immune checkpoints on immune cells (e.g., PD-1 and CTLA-4 on CAR-T cells) with their ligands (e.g., PD-L1 on tumor or stromal cells) transmits signals that directly suppress T cell activation and effector functions. The metabolic competition for essential nutrients (e.g., glucose and amino acids) between immune cells (T cells) and tumor cells or suppressive cells (MDSCs) is also shown. Additionally, physical obstacles that impede immune cell infiltration and contact tumor cells, including a dense extracellular matrix (ECM) and an abnormal tumor-associated vascular system that is dysfunctional and challenging for immune cells to extravasate through, are depicted. TGF-β, transforming growth factor-β; IL-10, interleukin-10; IL-4, interleukin-4; PD-1, programmed cell death protein 1; CTLA-4, cytotoxic T lymphocyte-associated protein 4; PD-L1, programmed death-ligand 1. Created in https://BioRender.com.

The TME represents a major biological barrier to CAR-T cell efficacy, particularly in solid tumors. This complex environment consists of immunosuppressive cells, inhibitory cytokines, metabolic barriers, a dense ECM, and cancer-associated fibroblasts (CAFs). This severely prevents CAR-T cells from entering tumor lesions, achieving effective activation, and maintaining sustained functionality (Fig. 4). Consequently, this constitutes a critical bottleneck that substantially restricts the effectiveness of CAR-T therapies against solid malignancies [40,41].

#### Immunosuppressive cells

Myeloid-derived suppressor cells (MDSCs), regulatory T cells (Tregs), and tumor-associated macrophages (TAMs, especially M2 macrophages) accumulate in large quantities in the TME and directly impede the activity and effectiveness of CAR-T cells through the secretion of inhibitory factors. Recent studies have revealed that cholesterol efflux from M2 macrophages suppresses CAR-T cell cytotoxicity by inducing immunosuppression in CD8^+^ T cells, ultimately driving them toward functional exhaustion [42]. This highlights how the metabolic programming of stromal cells can directly dictate CAR-T cell fitness.

#### Immunosuppressive cytokines and immune checkpoints

Beyond cellular interactions, the TME is saturated with immunosuppressive cytokines such as TGF-β, IL-10, and IL-4, which directly impair T cell activation and effector functions. Concurrently, tumors exploit multiple immune checkpoint pathways, including PD-1, CTLA-4, and TIGIT, to deliver potent inhibitory signals that dampen the CAR-T cell response [43]. This suppression is often mediated through ligand–receptor interactions, such as the binding of PD-L1, which is highly expressed on tumor or stromal cells, to PD-1 on CAR-T cells. The convergence of soluble cytokine-mediated suppression and checkpoint signaling creates a powerful, multi-layered inhibitory circuit.

#### Metabolic competition

A defining feature of the TME is its metabolic competition. In the TME, rapidly proliferating tumor cells and immunosuppressive cells consume nutrients in large quantities such as glucose and amino acids and produce metabolic wastes such as lactic acid and adenosine. The core metabolic pathways of T cells, namely, glycolysis, oxidative phosphorylation (OXPHOS), and fatty acid oxidation (FAO), are dynamically reconfigured during activation and differentiation [44]. Upon activation via antigen engagement, signaling through the phosphoinositide 3-kinase (PI3K)/protein kinase B (AKT)/mammalian target of rapamycin (mTOR) pathway up-regulates glucose transporter GLUT1 and promotes a metabolic shift toward glycolysis to meet the energetic demands of effector cells. However, within the TME, they face direct competition with tumor cells for the limited glucose supply. Furthermore, hypoxia suppresses mitochondrial OXPHOS, and acidosis alongside elevated reactive oxygen species (ROS) exacerbates T cell exhaustion. Additional suppression comes from metabolites like adenosine (via A₂A receptor signaling) and products of indoleamine 2,3-dioxygenase (IDO) activity. Thus, the failure to execute proper metabolic programming is a key resistance mechanism, making strategies to counteract these pathways vital for improving outcomes.

#### Physical barriers

The efficacy of CAR-T cells is further limited by the physical barriers, including the ECM and the abnormal tumor-associated vascular system. In solid tumors, 4 key physical characteristics, including increased solid stress, increased interstitial fluid pressure, increased macroscopic stiffness, and abnormal matrix microarchitecture, may impair the performance of infused CAR-T cells [45]. These barriers ensure that even functionally competent cells may never reach their intended targets, representing the first and most direct obstacle to successful therapy.

### Special mechanism of CAR-NK cell therapy

While CAR-NK cells share common challenges with CAR-T cells, including antigen escape, low persistence, low tumor-homing efficiency, and an immunosuppressive environment [46,47], they also face unique biological constraints rooted in NK cell physiology. A thorough understanding of these distinct mechanisms is critical for developing tailored strategies to enhance CAR-NK efficacy.

The translational potential of CAR-NK therapy is fundamentally constrained by the short lifespan in vivo. Without cytokine support, their functional persistence is typically limited to 1 to 2 weeks, a timeframe often inadequate for establishing durable disease control and predisposing to early relapse. This challenge is further compounded by donor-specific limitations, as allogeneic CAR-NK cells face potential rejection by host T cells recognizing non-self human leukocyte antigen (HLA) molecules, significantly reducing their persistence in clinical settings and necessitating the development of novel rejection-mitigation strategies. In addition, the manufacturing process itself presents a unique hurdle. When NK cells are expanded in vitro, the Fas/FasL axis and NKG2D receptors may recognize self-ligands, or CARs may recognize self-antigens, resulting in self-kill and affecting the number of cells ultimately obtained [48].

Furthermore, recent research has identified metabolic adaptation failure as a key mechanism of tumor relapse after CAR-NK therapy, characterized by an exhausted, dysfunctional phenotype. Single-cell transcriptomic analyses in preclinical models reveal that this failure encompasses specific deficits in core metabolic pathways, including diminished glycolytic capacity, impaired OXPHOS, and dysregulated fatty acid metabolism. Highly metabolically active tumor cells outcompete CAR-NK cells for nutrients, leading to energy deprivation, mitochondrial dysfunction, and consequent functional exhaustion that enables relapse [49]. CAR-NK cells are strongly inhibited by immunosuppressive factors in the TME, resulting in decreases in their cytotoxicity and cytokine secretion ability [46]. They are highly susceptible to TGF-β-mediated suppression, which directly attenuates their cytotoxicity, cytokine production, and expansion. This effect is synergistically enhanced by IL-10 from MDSCs, which activates the SOCS3 pathway to inhibit perforin and granzyme B synthesis [48]. Therefore, for CAR-NK cell therapy, maintaining the self-cell expansion and sustained function is of utmost importance to reduce drug resistance and recurrence.

Although NK cells naturally express a broad repertoire of chemokine receptors (including CXCR3, CXCR4, CCR5, and CCR7) that theoretically provide superior homing capability compared to T cells, this advantage is often negated in the complex solid TME. Together with the physical barrier formed by the fibrotic matrix, as a result, only 15% to 20% of the infused cells can penetrate into the tumor core [50]. This nonuniform distribution of infiltrating cells enables the survival of antigen-negative or low-expression tumor clones in poorly accessed regions, ultimately driving antigen-escape recurrence through selective pressure. Collectively, addressing these interconnected challenges through improved persistence engineering, enhanced homing strategies, and immune environment modification will be essential for unlocking the full potential of CAR-NK immunotherapy.

## Response Strategies and Research Progress

**Fig. 5.**
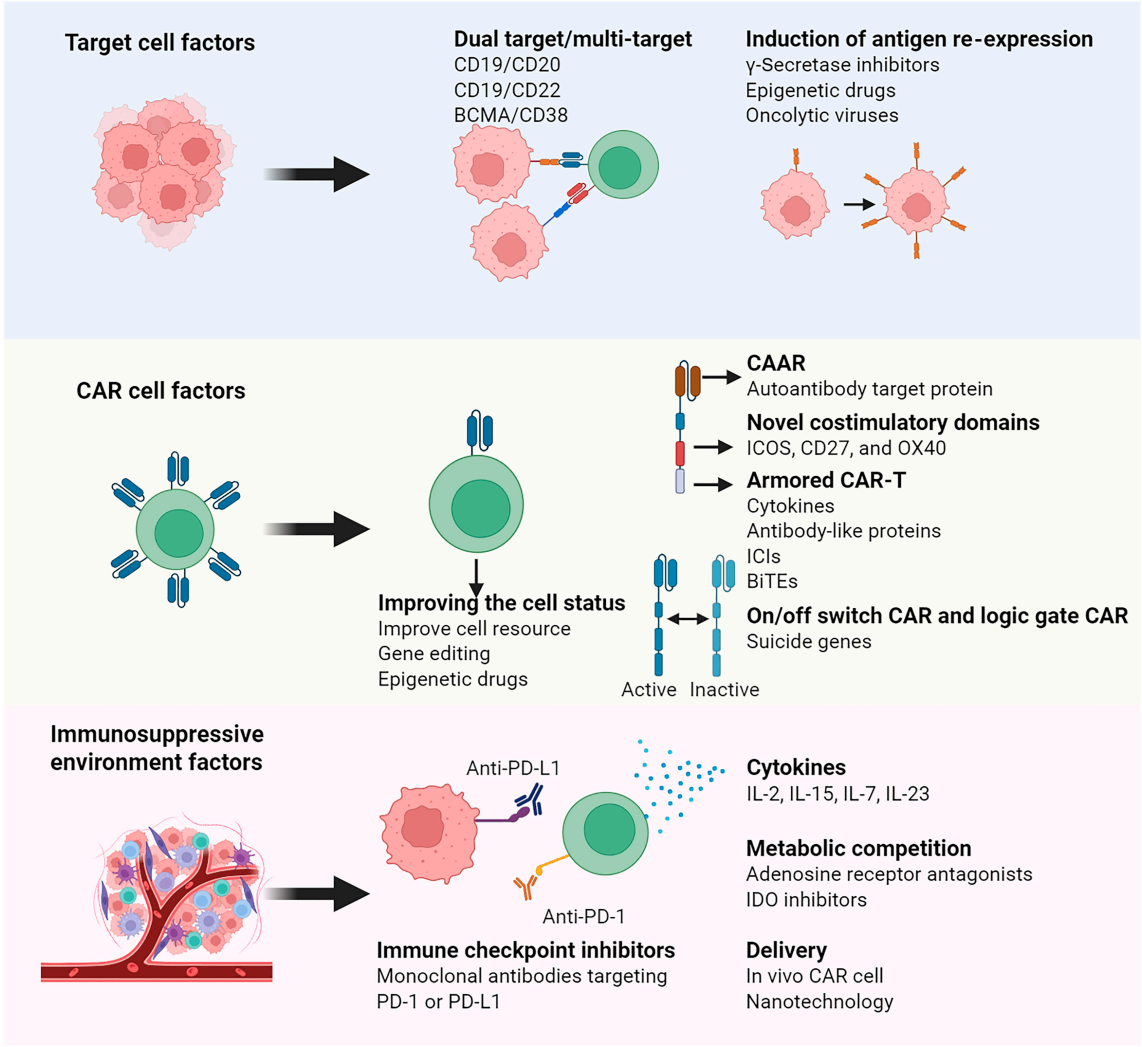
Three key factors contributing to resistance and recurrence, along with their corresponding strategies. This diagram outlines the primary strategies being developed to combat resistance and recurrence, which are divided into 3 categories: target cell factors, CAR-T cell factors, and immunosuppressive environment factors. CAR, chimeric antigen receptor; BCMA, B cell maturation antigen; ICOS, inducible T cell costimulatory; OX40 (also known as CD134), a TNF receptor family costimulatory molecule; ICI, immune checkpoint inhibitor; PD-1, programmed cell death protein 1; PD-L1, programmed death-ligand 1; BITE, bispecific T cell engager; CAAR, chimeric autoantibody receptor. Created in https://BioRender.com.

The interactions among target cells, the CAR cell product, and the immunosuppressive environment collectively affect the final therapeutic outcome. Overcoming these challenges requires a comprehensive strategy, which has become a key focus of current research (Fig. 5).

### Strategies for the target

#### Dual-target/multitarget CARs

To combat antigenic escape, researchers are developing combination strategies that target 2 or more antigens simultaneously or sequentially. This can be achieved by combining 2 scFvs with the same CAR or by infusing 2 CAR-T cells targeting different antigens. This sequential multi-antigen targeting paradigm, clinically implemented as cocktail therapy, translates into proven safety and enhanced efficacy for refractory B cell lymphoma patients, with marked improvement in long-term response maintenance [51–53]. Breakthroughs have also emerged in treating central nervous system lymphomas [54] and glioblastoma multiforme [55]. Currently, numerous new cocktail therapies targeting diverse antigens are being explored [56]. CD19/CD22 CAR-T cell therapies are commonly used to treat B-ALL and large B cell lymphoma (LBCL), resulting in high remission rates and preventing antigen-negative relapse [57–59]. BCMA/GPRC5D or BCMA/CD38 CAR-T cells have shown remarkable efficacy in treating MM patients resistant to BCMA CAR-T cells [60,61]. The application of this strategy is also expanding into autoimmune diseases, where dual targeting of CD19 and BCMA has demonstrated promising remission outcomes [62]. Future research will concentrate on discovering more specific antigens with stable expression characteristics and translating these multi-targeting constructs into broader clinical practice.

#### Induction of antigen re-expression

Complementary to multi-targeting, a pharmacologic strategy seeks to reverse antigen escape by forcibly re-establishing the target on the target cell surface. Small-molecule agents can modulate cellular pathways to up-regulate or stabilize target antigen density, thereby resensitizing tumor cells to CAR-mediated killing. In MM treatment, γ-secretase inhibitors prevent BCMA cleavage and shedding, significantly increasing its surface density and enhancing the activity of anti-BCMA CAR-T cells in preclinical models [63], with clinical trial studies now underway [64]. Combined epigenetic drugs also increase the immunogenicity of target cells [65]. For example, in preclinical evaluation, bryostatin 1 treatment effectively up-regulates CD22 surface density, resulting in enhanced performance of CAR-T cell treatments designed to recognize this target [66]. Beyond small molecules, oncolytic viruses (OVs) can activate immune response and modifying OVs to express specific CAR antigens and deliver them to the tumor surface can enhance the targeting of corresponding CAR-T cells, demonstrating considerable potential for solid tumor applications [67–69]. Collectively, these approaches aim to remodel the tumor cell surface to make it a more visible and vulnerable target for cellular immunotherapy.

### Optimization of the CAR-T cell strategy

#### Optimizing the CAR structure design

The pursuit of next-generation CAR-T cells centers on functionality, safety, and controllability. Novel CMs, including ICOS, CD27, and OX40, are currently undergoing evaluation, with some demonstrating potential in enhancing persistence, regulating cellular metabolism, and improving immune tolerance [70–73]. Chimeric autoantibody receptor-T (CAAR-T) cells are produced to attack B cells that produce target antibodies [74], which have undergone increased development, with increasing numbers of pathogenic antibodies being discovered [75]. A critical frontier in CAR design is the incorporation of built-in control systems. On/off switch CAR and logic gate CAR (AND, OR, NOT gates) not only help overcome drug resistance but also increase safety and precision. Suicide genes including herpes simplex virus–thymidine kinase (HSV-TK), inducible caspase 9 (iCasp9), and CD20 have been introduced as safety switches [76,77]. Pharmacological agents including specific tyrosine kinase inhibitors like dasatinib have demonstrated capacity for precise regulation of CAR-T cell activity [78]. To transcend the limitations of conventional CAR-T cells, armored CAR-T cells are engineered to express various proteins, such as cytokines (such as IL-12, IL-18, and IL-7), antibody-like proteins, immune checkpoint inhibitors (ICIs) (such as PD-1, CTLA4, and NKG2A), and bispecific T cell engagers (BiTEs), which help induce a memory phenotype, target antigens, and remodel the immunosuppressive microenvironment [79–81]. For example, CAR-T cells that secrete IL-10 promoted the proliferation and effector functions of CAR-T cells in preclinical models [82]. With their unique ability to engage CD3 on T cells while selectively targeting B lymphocytes, BiTEs present new opportunities for treating autoimmune conditions [83–85]. In addition, by designing “universal” CAR constructs that target different antigens and producing corresponding universal CAR-T cells, these cells can be implemented as “off-the-shelf” therapeutics for large-scale clinical applications [86]. This holistic approach to receptor design is thus creating a new generation of smarter, safer, and more versatile cellular drugs.

#### Improving the cell status and functions

The functional efficacy of CAR-T cells is profoundly influenced by their initial differentiation state and metabolic fitness, making the optimization of the cellular product itself as important as the design of its receptor. To improve the cell status, T cells with stronger self-renewal and durable survival abilities like naive T cells and stem cell memory T cells are preferred [87]. The timing of cell collection is also critical, as clinical outcomes are superior when T cell collection occurs at disease onset rather than after multiple lines of therapy [23]. Pharmacologic interventions are being leveraged to reverse or prevent functional exhaustion. Combined epigenetic drugs increase the immunogenicity of target cells while boosting CAR-T cell performance by preventing or reversing cellular exhaustion. Multiple clinical investigations have employed inhibitors targeting DNMT, HDAC, and EZH2 in combined therapeutic approaches [30,88,89]. Treatment with drugs such as dasatinib can induce resting through forced CAR protein down-regulation, facilitating the development of memory-like characteristics alongside comprehensive transcriptomic and epigenetic remodeling in previously exhausted CAR-T populations [90]. Recent advances have identified additional pathways for functional enhancement, as demonstrated by the VEGF inhibitor axitinib, which activates canonical Wnt/β-catenin signaling to reverse CAR-T cell differentiation and exhaustion during manufacturing [91]. This positions the maintenance of stemness-associated pathways as a promising strategy for sustaining CAR cell proliferative capacity.

Additionally, to specifically degrade the ECM in solid tumors, an engineering approach that incorporates chemokine receptors (including CXCR1, CXCR2, and CXCR4) into CAR-T cells enhances their migration capacity [92]. Ultimately, by concurrently optimizing the intrinsic quality of the T cell product and its capacity to navigate physiological obstacles, these strategies aim to generate cellular therapies that are not only potent upon infusion but also endowed with the durability to maintain long-term disease control.

### Strategies to modify the immunosuppressive environment

Direct disruption of inhibitory signaling pathways represents a cornerstone strategy for restoring CAR cell efficacy. Combined ICIs can cause significant antitumor activity. For example, monoclonal antibodies (mAbs) targeting PD-1 or PD-L1 demonstrate capacity to improve the functional state of tumor-infiltrating lymphocytes, reinvigorating their inherent capacity to identify and eliminate cancer cells [93]. This approach extends to novel agents such as TIM-3 palmitoylation inhibitors that strengthen immune responses mediated by CAR-T cells and NK cells [94], and S1PR3 antagonists that remodel the TME by promoting macrophage activation and polarization toward the proinflammatory phenotype [95]. Furthermore, targeted immune checkpoint inhibition can be achieved through CAR and gene editing methods, such as BCMA CAR-T cells carrying anti-PD-1 short hairpin RNA (shRNA) boxes [96]. However, the safety and adverse effects of these therapies require careful monitoring [97].

Overcoming the cytokine deficiency and metabolic stress is equally critical. Cytokines are utilized to support both persistence and proliferation of CAR-T cells. According to various studies, proliferative cytokines (such as IL-2 and IL-15) increase T cell activity, while IL-7 promotes the steady-state expansion of both native and memory T cells, and IL-23 facilitates the proliferation and survival of T cells [80,98]. To overcome the problem of metabolic competition, adenosine receptor antagonists and IDO inhibitors can be used to improve the metabolic status of the TME. Adenosine serves as the primary mediator of immune suppression in hypoxic TMEs. In preclinical studies, CRISPR/Cas9-mediated adenosine A_2A_ receptor (A_2A_R) deletion has been found to strengthen CAR-T cell effector responses [99].

To overcome the insufficient CAR-T cell infiltration and inhibitory effects of the TME, innovative in vivo CAR-T cell technology delivers CAR genes directly into host T cells via in vivo delivery systems for in situ reprogramming [100]. This strategy improves CAR-T cell adaptability to the tumor environment and avoids systemic barriers. Current research emphasizes the development of innovative delivery strategies, including regional perfusion and localized delivery [101]. As an emerging and promising method, nanotechnology also offers promise by enabling targeted delivery, remodeling the TME, and supporting the persistence and function of CAR-T cell in solid tumors [102,103].

### Special strategies for CAR-NK cells

While CAR-NK cells benefit from established strategies such as multi-targeting and checkpoint inhibition, their unique biology necessitates tailored approaches that optimize their innate advantages [104]. The design of optimized NK cell-specific CARs, such as CD3ζ, DAP10, and DAP12, is under investigation [105–107]. The use of NK cell-activatable receptors and their downstream adapter proteins to replace T cell elements to form NK-specific CARs can greatly improve their signal transduction and cell activation abilities [108].

To ensure sustained functionality, cytokine engineering is the core strategy for improving the function of CAR-NK cells. FT596 [an induced pluripotent stem cell (iPSC)-derived ready-to-use CD19 CAR-NK cell] expressing recombinant IL-15 and IL-15 receptor α (IL-15 RF) promotes their proliferation and enhances persistence. Recently, evidence suggests that deleting the transcription factor cyclic adenosine monophosphate (AMP) response element modulator (CREM), a pivotal regulator of NK cell function, enhances CAR-NK cell effector activity and resistance to tumor immunosuppression, positioning it as a promising therapeutic target [109]. Another approach to prevent NK cell depletion involves generating immune cells with a memory-like phenotype. Studies have established that cytokine-induced memory-like (CIML) NK cells, generated through in vitro induction via the cytokines IL-12, IL-15, and IL-18, have demonstrated long-term tumor cell persistence and cytotoxicity in preclinical and clinical trials [104].

For solid tumors, modifying CAR-NK cells through gene editing to continuously secrete IL-15/IL-21 or express chemokine receptors such as CCR2 and CXCR4 significantly strengthens their tumor-specific trafficking and infiltration potential [110]. Furthermore, the functional persistence of CAR-NK cells in vivo is limited and may require more frequent administration to improve survival and prolong the antitumor response [111]. The exploration of novel targets is also yielding promising results, with the death receptor 5 (DR5) pathway emerging as a compelling candidate [112]. This approach is further enhanced by small molecules like Bortisitan, which up-regulates DR5 to synergize with CAR-NK cells via TRAIL-mediated apoptosis, effectively overcoming therapy resistance [113].

To further improve the antibody-dependent cellular cytotoxicity (ADCC) effect of NK cells, researchers are exploring methods of combining mAbs that can mediate ADCC with genetically engineered NK cells to enhance anticancer activity [114]. Finally, addressing manufacturing challenges through advanced cryopreservation techniques ensures better cell viability and function, thereby solidifying the foundation for reliable “off-the-shelf” CAR-NK products [115,116]. Collectively, these specialized strategies are poised to unlock the full potential of CAR-NK cell therapy across a broad spectrum of malignancies.

### Combination treatment strategies

It is difficult to completely overcome the problems of drug resistance and relapse by relying on CAR-T cell or CAR-NK cell therapy alone. By integrating these cellular therapies with chemotherapy, radiotherapy, targeted therapy, and other therapies, a synergistic superimposed effect can be produced. The combination of CAR-T cells and CAR-NK cells overcomes the limitations of single-cell therapy through functional complementarity. Given the demonstrated efficacy of CAR-NK cells in cases where CAR-T therapy has failed, a sequential scheme was designed for CAR-NK cells to rapidly reduce the load before the CAR-T cells establish long-term memory [115], which achieved a 12-month disease-free survival rate (DFS) of 75% in relapsed or refractory (R/R) B-ALL patients, which was significantly greater than that of monotherapy [117]. Further innovation includes iPSC-derived NK-T hybrid cells, which enable standardized production of dual CAR products and combine the immediate cytotoxicity of NK cells with the long-term memory capability of T cells while reducing manufacturing costs by 40% [115]. The combination of traditional treatment methods can create more favorable conditions for cell therapy. Combined chemotherapy may amplify antitumor efficacy or work synergistically with CAR-T cells to eliminate tumors [118,119]. The combination of high-dose chemotherapy and autologous stem cell transplantation (HDT/ASCT) has shown outstanding results in treating relapsed/refractory LBCL [120]. Radiotherapy locally destroys tumors, modifies the immunosuppressive microenvironment, and enhances CAR-T cell infiltration and function [121–123]. In autoimmune diseases, combined chemotherapy serves as a pretreatment for lymphocyte depletion before CAR-T cell infusion, creating optimal conditions for cell implantation and expansion. When used alongside glucocorticoids, it acts as a bridging strategy before CAR-T cell administration [124]. Additionally, studies on various vaccine models, including mRNA-, peptide-, viral vector-, and dendritic cell (DC)-based vaccines, are underway. Tumor vaccines stimulate endogenous immune responses and can enhance CAR-T cell functionality in individuals receiving dialysis therapy [125].

## Challenges and Future Prospects

The core breakthrough in future development lies in empowering cells with the ability to sense complex environmental cues, make decisions based on integrated logic, communicate with the host immune system, and adapt dynamically to the evolving disease landscape.

Future CAR cells will be designed as “sensing” entities that perceive complex pathological cues beyond single antigens. By integrating synthetic biology, CAR cells can be programmed to respond to dynamic immunosuppressive environment cues like hypoxia, acidosis, or specific enzyme activity. This contextual awareness ensures that potent cytotoxicity is precisely confined to pathological niches, substantially enhancing therapeutic safety. Advancements in “decision-making” will enable sophisticated target discrimination through Boolean logic gates [126]. AND-gate CARs requiring dual antigen recognition minimize on-target/off-tumor toxicity, while NOT-gates can deliver veto signals upon encountering healthy tissue markers. This evolves into complex “IF-THEN” programs where cells activate fully only when multiple conditions are met, transforming them into discerning therapeutic agents. Aimed at “communicating” with the host immune system, engineering CAR cells to secrete immunomodulators or bispecific engagers can recruit and activate endogenous immune cells like DCs and T cells. This remodels the immunosuppressive TME into an immunostimulatory one, fostering a broad, coordinated, and durable immune response against heterogeneous tumors. The ultimate goal involves creating “adaptive” therapies capable of co-evolving with dynamic diseases. Strategies include designing circuits that confer a selective proliferation advantage to CAR clones upon successful target engagement, or infusing diverse CAR libraries that allow the most effective clones to expand in vivo. This creates an adaptive therapeutic ecosystem capable of countering antigen escape. Concurrently, manufacturing innovations are paving the way for universally applicable off-the-shelf products, significantly reducing costs and expanding accessibility [127]. As these technological advances mature, CAR-T and CAR-NK platforms are poised to expand beyond oncology and autoimmunity into chronic infections and probably age-related diseases. The convergence of sensing, logic, communication, and adaptation capabilities will ultimately establish cell therapies as precise, scalable, and universal modalities for targeting pathological cells while preserving healthy tissues.

Overall, drug resistance and the recurrence of CAR-T and CAR-NK cell therapy are severe challenges in treatment. The mechanism is complex and involves target cells, CAR-T and CAR-NK cells, the immunosuppressive environment, and many other aspects. Current strategies are transitioning from traditional single approaches to multidimensional, combined comprehensive solutions. Future efforts will focus on making cell therapies smarter, more precise, efficient, and safer. Through combination therapies and mechanistic exploration, we aim to ultimately overcome challenges in treating cancers and autoimmune diseases, expand their application scope, and benefit more patients.

## References

[1] Liu Y, Duan Y, Du Z, Lu B, Liu S, Li L, Tian M, Li L, Yao R, Ouyang C, et al. Current challenges and emerging opportunities of chimeric antigen receptor-engineered cell immunotherapy. Exp Hematol Oncol. 2025;14(1):92. 40604935 10.1186/s40164-025-00683-y PMC12224691

[2] Gu R, Shen J, Zhang J, Mao J, Ye Q. Revolutionizing autoimmune kidney disease treatment with chimeric antigen receptor-T cell therapy. Research. 2025;8:0712. 40405911 10.34133/research.0712 PMC12095914

[3] Valtis YK, Chin KK, Nemirovsky D, Devlin SM, Yisraeli Salman M, Boussi L, Cadzin B, McLoughlin C, Cathcart E, Davis P, et al. Barriers to chimeric antigen receptor T-cell therapy. JAMA Oncol. 2025;11(7):781–784. 40372728 10.1001/jamaoncol.2025.1127 PMC12082480

[4] Moscarelli J, Zahavi D, Maynard R, Weiner LM. The next generation of cellular immunotherapy: Chimeric antigen receptor-natural killer cells. Transplant Cell Ther. 2022;28(10):650–656. 35788086 10.1016/j.jtct.2022.06.025 PMC9547868

[5] Wang W, Liu Y, He Z, Li L, Liu S, Jiang M, Zhao B, Deng M, Wang W, Mi X, et al. Breakthrough of solid tumor treatment: CAR-NK immunotherapy. Cell Death Discov. 2024;10(1):40. 38245520 10.1038/s41420-024-01815-9 PMC10799930

[6] Westin JR, Oluwole OO, Kersten MJ, Miklos DB, Perales MA, Ghobadi A, Rapoport AP, Sureda A, Jacobson CA, Farooq U, et al. Survival with axicabtagene ciloleucel in large B-cell lymphoma. N Engl J Med. 2023;389(2):148–157. 37272527 10.1056/NEJMoa2301665

[7] Fujiwara K, Tsunei A, Kusabuka H, Ogaki E, Tachibana M, Okada N. Hinge and transmembrane domains of chimeric antigen receptor regulate receptor expression and signaling threshold. Cells. 2020;9(5):1182. 32397414 10.3390/cells9051182 PMC7291079

[8] Mace EM. Human natural killer cells: Form, function, and development. J Allergy Clin Immunol. 2023;151(2):371–385. 36195172 10.1016/j.jaci.2022.09.022 PMC9905317

[9] Yang S, Cao B, Zhou G, Zhu L, Wang L, Zhang L, Kwok HF, Zhang Z, Zhao Q. Targeting B7-H3 immune checkpoint with chimeric antigen receptor-engineered natural killer cells exhibits potent cytotoxicity against non-small cell lung cancer. Front Pharmacol. 2020;11:1089. 32848731 10.3389/fphar.2020.01089 PMC7406658

[10] Shah NN, Fry TJ. Mechanisms of resistance to CAR T cell therapy. Nat Rev Clin Oncol. 2019;16(6):372–385. 30837712 10.1038/s41571-019-0184-6 PMC8214555

[11] Cao LY, Zhao Y, Chen Y, Ma P, Xie JC, Pan XM, Zhang X, Chen YC, Wang Q, Xie LL. CAR-T cell therapy clinical trials: Global progress, challenges, and future directions from ClinicalTrials.gov insights. Front Immunol. 2025;16:1583116. 40463393 10.3389/fimmu.2025.1583116 PMC12129935

[12] Schett G, Mackensen A, Mougiakakos D. CAR T-cell therapy in autoimmune diseases. Lancet. 2023;402(10416):2034–2044. 37748491 10.1016/S0140-6736(23)01126-1

[13] Lin H, Yang X, Ye S, Huang L, Mu W. Antigen escape in CAR-T cell therapy: Mechanisms and overcoming strategies. Biomed Pharmacother. 2024;178: Article 117252. 39098176 10.1016/j.biopha.2024.117252

[14] Asnani M, Hayer KE, Naqvi AS, Zheng S, Yang SY, Oldridge D, Ibrahim F, Maragkakis M, Gazzara MR, Black KL, et al. Retention of CD19 intron 2 contributes to CART-19 resistance in leukemias with subclonal frameshift mutations in CD19. Leukemia. 2020;34(4):1202–1207. 31591467 10.1038/s41375-019-0580-z PMC7214268

[15] Orlando EJ, Han X, Tribouley C, Wood PA, Leary RJ, Riester M, Levine JE, Qayed M, Grupp SA, Boyer M, et al. Genetic mechanisms of target antigen loss in CAR19 therapy of acute lymphoblastic leukemia. Nat Med. 2018;24(10):1504–1506. 30275569 10.1038/s41591-018-0146-z

[16] Ruella M, Xu J, Barrett DM, Fraietta JA, Reich TJ, Ambrose DE, Klichinsky M, Shestova O, Patel PR, Kulikovskaya I, et al. Induction of resistance to chimeric antigen receptor T cell therapy by transduction of a single leukemic B cell. Nat Med. 2018;24(10):1499–1503. 30275568 10.1038/s41591-018-0201-9 PMC6511988

[17] Chen Y, Xin Q, Zhu M, Qiu J, Qiu J, Li R, Tu J. Trogocytosis in CAR immune cell therapy: A key mechanism of tumor immune escape. Cell Commun Signal. 2024;22(1):521. 39468646 10.1186/s12964-024-01894-2 PMC11514842

[18] Hamieh M, Dobrin A, Cabriolu A, van der Stegen SJC, Giavridis T, Mansilla-Soto J, Eyquem J, Zhao Z, Whitlock BM, Miele MM, et al. CAR T cell trogocytosis and cooperative killing regulate tumour antigen escape. Nature. 2019;568(7750):112–116. 30918399 10.1038/s41586-019-1054-1 PMC6707377

[19] Zhai Y, Du Y, Li G, Yu M, Hu H, Pan C, Wang D, Shi Z, Yan X, Li X, et al. Trogocytosis of CAR molecule regulates CAR-T cell dysfunction and tumor antigen escape. Signal Transduct Target Ther. 2023;8(1):457. 38143263 10.1038/s41392-023-01708-w PMC10749292

[20] Jacoby E, Nguyen SM, Fountaine TJ, Welp K, Gryder B, Qin H, Yang Y, Chien CD, Seif AE, Lei H, et al. CD19 CAR immune pressure induces B-precursor acute lymphoblastic leukaemia lineage switch exposing inherent leukaemic plasticity. Nat Commun. 2016;7:12320. 27460500 10.1038/ncomms12320 PMC4974466

[21] Silbert SK, Rankin AW, Hoang CN, Semchenkova A, Myers RM, Zerkalenkova E, Wang HW, Kovach AE, Yuan CM, Delgado Colon D, et al. Project EVOLVE: An international analysis of postimmunotherapy lineage switch, an emergent form of relapse in leukemia. Blood. 2025;146(4):437–455. 40193715 10.1182/blood.2024026655 PMC12333221

[22] Meacham CE, Morrison SJ. Tumour heterogeneity and cancer cell plasticity. Nature. 2013;501(7467):328–337. 24048065 10.1038/nature12624 PMC4521623

[23] Yang Z, Ha B, Wu Q, Ren F, Yin Z, Zhang H. Expanding the horizon of CAR T cell therapy: From cancer treatment to autoimmune diseases and beyond. Front Immunol. 2025;16:1544532. 40046061 10.3389/fimmu.2025.1544532 PMC11880241

[24] Wang M, DeStefano VM, Ding L, Hong M, Zeng R, Wada M, Pinz K, Chow JE, Hershkowitz N, Wang M, et al. Validation of BCMA-CD19 compound CAR-T therapy in SLE overlap syndrome: Over 1.5-year follow-up. Stem Cell Rev Rep. 2025;21(6):1750–1759. 40549291 10.1007/s12015-025-10923-7

[25] Huang Y, Qin Y, He Y, Qiu D, Zheng Y, Wei J, Zhang L, Yang DH, Li Y. Advances in molecular targeted drugs in combination with CAR-T cell therapy for hematologic malignancies. Drug Resist Updat. 2024;74: Article 101082. 38569225 10.1016/j.drup.2024.101082

[26] Kimman T, Cuenca M, Tieland RG, Rockx-Brouwer D, Janssen J, Motais B, Slomp A, Pleijte C, Heijhuurs S, Meringa AD, et al. Engineering anti-BCMA CAR T cells for enhancing myeloma killing efficacy via apoptosis regulation. Nat Commun. 2025;16(1):4638. 40389394 10.1038/s41467-025-59818-8 PMC12089368

[27] Tang L, Zhang H, Zhou F, Wei Q, Du M, Wu J, Li C, Luo W, Zhou J, Wang X, et al. Targeting autophagy overcomes cancer-intrinsic resistance to CAR-T immunotherapy in B-cell malignancies. Cancer Commun. 2024;44(3):408–432. 10.1002/cac2.12525 PMC10958674 38407943

[28] Zebley CC, Youngblood B. Mechanisms of T cell exhaustion guiding next-generation immunotherapy. Trends Cancer. 2022;8(9):726–734. 35570136 10.1016/j.trecan.2022.04.004 PMC9388609

[29] Xia A, Zhang Y, Xu J, Yin T, Lu XJ. T cell dysfunction in cancer immunity and immunotherapy. Front Immunol. 2019;10:1719. 31379886 10.3389/fimmu.2019.01719 PMC6659036

[30] Wang K, Ou K, Zeng Y, Yue C, Zhuo Y, Wang L, Chen H, Tu S. Epigenetic landscapes drive CAR-T cell kinetics and fate decisions: Bridging persistence and resistance. Crit Rev Oncol Hematol. 2025;211: Article 104729. 40246258 10.1016/j.critrevonc.2025.104729

[31] Ponzo M, Drufuca L, Buracchi C, Sindoni MM, Nucera S, Bugarin C, Bason R, Rossetti G, Bonnal R, Meli C, et al. Acquisition of an immunosuppressive microenvironment after anti-CD19 CAR T-cell therapy is associated with T-cell dysfunction and resistance. J Immunother Cancer. 2025;13(10): Article e011768. 41135951 10.1136/jitc-2025-011768 PMC12557794

[32] Rakhshandehroo T, Mantri SR, Moravej H, Louis BBV, Salehi Farid A, Munaretto L, Regan K, Khan RMM, Wolff A, Farkash Z, et al. A CAR enhancer increases the activity and persistence of CAR T cells. Nat Biotechnol. 2025;43(6):948–959. 39079964 10.1038/s41587-024-02339-4 PMC11779983

[33] Turtle CJ, Hanafi LA, Berger C, Gooley TA, Cherian S, Hudecek M, Sommermeyer D, Melville K, Pender B, Budiarto TM, et al. CD19 CAR-T cells of defined CD4+:CD8+ composition in adult B cell ALL patients. J Clin Invest. 2016;126(6):2123–2138. 27111235 10.1172/JCI85309 PMC4887159

[34] Khalifeh M, Salman H. Engineering resilient CAR T cells for immunosuppressive environment. Mol Ther. 2025;33(6):2391–2405. 39863931 10.1016/j.ymthe.2025.01.035 PMC12172186

[35] Ledergor G, Fan Z, Wu K, McCarthy E, Hyrenius-Wittsten A, Starzinski A, Chang H, Bridge M, Kwek S, Cheung A, et al. CD4+ CAR T-cell exhaustion associated with early relapse of multiple myeloma after BCMA CAR T-cell therapy. Blood Adv. 2024;8(13):3562–3575. 38574299 10.1182/bloodadvances.2023012416 PMC11319832

[36] Scholler N, Perbost R, Locke FL, Jain MD, Turcan S, Danan C, Chang EC, Neelapu SS, Miklos DB, Jacobson CA, et al. Tumor immune contexture is a determinant of anti-CD19 CAR T cell efficacy in large B cell lymphoma. Nat Med. 2022;28(9):1872–1882. 36038629 10.1038/s41591-022-01916-x PMC9499856

[37] Kawalekar OU, O’Connor RS, Fraietta JA, Guo L, McGettigan SE, Posey AD Jr, Patel PR, Guedan S, Scholler J, Keith B, et al. Distinct signaling of coreceptors regulates specific metabolism pathways and impacts memory development in CAR T cells. Immunity. 2016;44(2):380–390. 26885860 10.1016/j.immuni.2016.01.021 PMC13498396

[38] Cook MS, King E, Flaherty KR, Siddika K, Papa S, Benjamin R, Schurich A. CAR-T cells containing CD28 versus 4-1BB co-stimulatory domains show distinct metabolic profiles in patients. Cell Rep. 2025;44(7): Article 115973. 40650909 10.1016/j.celrep.2025.115973

[39] Rossi M, Breman E. Engineering strategies to safely drive CAR T-cells into the future. Front Immunol. 2024;15:1411393. 38962002 10.3389/fimmu.2024.1411393 PMC11219585

[40] Tong L, Jiménez-Cortegana C, Tay AHM, Wickström S, Galluzzi L, Lundqvist A. NK cells and solid tumors: Therapeutic potential and persisting obstacles. Mol Cancer. 2022;21(1):206. 36319998 10.1186/s12943-022-01672-z PMC9623927

[41] Liu ZQ, Zhou ZK, Dang Q, Xu H, Lv JX, Li HY, Han XW. Immunosuppression in tumor immune microenvironment and its optimization from CAR-T cell therapy. Theranostics. 2022;12(14):6273–6290. 36168626 10.7150/thno.76854 PMC9475465

[42] Yan ZX, Dong Y, Qiao N, Zhang YL, Wu W, Zhu Y, Wang L, Cheng S, Xu PP, Zhou ZS, et al. Cholesterol efflux from C1QB-expressing macrophages is associated with resistance to chimeric antigen receptor T cell therapy in primary refractory diffuse large B cell lymphoma. Nat Commun. 2024;15(1):5183. 38890370 10.1038/s41467-024-49495-4 PMC11189439

[43] Toor SM, Sasidharan Nair V, Decock J, Elkord E. Immune checkpoints in the tumor microenvironment. Semin Cancer Biol. 2020;65:1–12. 31265893 10.1016/j.semcancer.2019.06.021

[44] Xia X, Yang Z, Lu Q, Liu Z, Wang L, Du J, Li Y, Yang DH, Wu S. Reshaping the tumor immune microenvironment to improve CAR-T cell-based cancer immunotherapy. Mol Cancer. 2024;23(1):175. 39187850 10.1186/s12943-024-02079-8 PMC11346058

[45] Luo Z, Yao X, Li M, Fang D, Fei Y, Cheng Z, Xu Y, Zhu B. Modulating tumor physical microenvironment for fueling CAR-T cell therapy. Adv Drug Deliv Rev. 2022;185: Article 114301. 35439570 10.1016/j.addr.2022.114301

[46] Khawar MB, Sun H. CAR-NK cells: From natural basis to design for kill. Front Immunol. 2021;12: Article 707542. 34970253 10.3389/fimmu.2021.707542 PMC8712563

[47] Wrona E, Borowiec M, Potemski P. CAR-NK cells in the treatment of solid tumors. Int J Mol Sci. 2021;22(11):5899. 34072732 10.3390/ijms22115899 PMC8197981

[48] Valeri A, García-Ortiz A, Castellano E, Córdoba L, Maroto-Martín E, Encinas J, Leivas A, Río P, Martínez-López J. Overcoming tumor resistance mechanisms in CAR-NK cell therapy. Front Immunol. 2022;13: Article 953849. 35990652 10.3389/fimmu.2022.953849 PMC9381932

[49] Li L, Mohanty V, Dou J, Huang Y, Banerjee PP, Miao Q, Lohr JG, Vijaykumar T, Frede J, Knoechel B, et al. Loss of metabolic fitness drives tumor resistance after CAR-NK cell therapy and can be overcome by cytokine engineering. Sci Adv. 2023;9(30): Article eadd6997. 37494448 10.1126/sciadv.add6997 PMC10371011

[50] Zhu X, Li Y, Liu H, Xiao Z. FN1 from cancer-associated fibroblasts orchestrates pancreatic cancer metastasis via integrin-PI3K/AKT signaling. Front Oncol. 2025;15:1595523. 40678072 10.3389/fonc.2025.1595523 PMC12267018

[51] Wei J, Mao Z, Wang N, Huang L, Cao Y, Sun W, Long X, Tan J, Li C, Xiao Y, et al. Long-term outcomes of relapsed/refractory double-hit lymphoma (r/r DHL) treated with CD19/22 CAR T-cell cocktail therapy. Clin Transl Med. 2020;10(5): Article e176. 10.1002/ctm2.176 PMC7507504 32997409

[52] Wang N, Hu X, Cao W, Li C, Xiao Y, Cao Y, Gu C, Zhang S, Chen L, Cheng J, et al. Efficacy and safety of CAR19/22 T-cell cocktail therapy in patients with refractory/relapsed B-cell malignancies. Blood. 2020;135(1):17–27. 31697824 10.1182/blood.2019000017

[53] Huang L, Li J, Yang J, Zhang X, Zhang M, He J, Zhang G, Li W, Wang H, Li J, et al. Safety and efficacy of humanized versus murinized CD19 and CD22 CAR T-cell cocktail therapy for refractory/relapsed B-cell lymphoma. Cells. 2022;11(24):4085. 36552849 10.3390/cells11244085 PMC9776474

[54] Zhou X, Yu Q, Dai Z, Wang J, Li C, Huang L, Zhang Y, Cao Y. CD19/CD22 CAR-T-cell cocktail therapy following autologous transplantation is an optimizing strategy for treating relapsed/refractory central nervous system lymphoma. Exp Hematol Oncol. 2024;13(1):100. 39397022 10.1186/s40164-024-00538-y PMC11471030

[55] Zhu G, Sun Z, Liu Y, Liu J, Guo L, Pei G, Jiang Y, Miao B, Li Z, Zhang P, et al. Rational design and organoid-based evaluation of a cocktail CAR-γδ T cell therapy for heterogeneous glioblastoma. Adv Sci. 2025;12(19): Article e2501772. 10.1002/advs.202501772 PMC12097020 40112194

[56] Nakatsura T, Takenouchi K, Kataoka J, Ito Y, Kikuchi S, Kinoshita H, Ohnuki K, Suzuki T, Tsukamoto N. Expression profiles of five common cancer membrane protein antigens collected for the development of cocktail CAR-T cell therapies applicable to most solid cancer patients. Int J Mol Sci. 2025;26(5):2145. 40076777 10.3390/ijms26052145 PMC11900252

[57] Ghorashian S, Lucchini G, Richardson R, Nguyen K, Terris C, Guvenel A, Oporto-Espuelas M, Yeung J, Pinner D, Chu J, et al. CD19/CD22 targeting with cotransduced CAR T cells to prevent antigen-negative relapse after CAR T-cell therapy for B-cell ALL. Blood. 2024;143(2):118–123. 37647647 10.1182/blood.2023020621

[58] Spiegel JY, Patel S, Muffly L, Hossain NM, Oak J, Baird JH, Frank MJ, Shiraz P, Sahaf B, Craig J, et al. CAR T cells with dual targeting of CD19 and CD22 in adult patients with recurrent or refractory B cell malignancies: A phase 1 trial. Nat Med. 2021;27(8):1419–1431. 34312556 10.1038/s41591-021-01436-0 PMC8363505

[59] Phely L, Hensen L, Faul C, Ruff CA, Schneider D, Bethge WA, Lengerke C. Allogeneic CD19/CD22 CAR T-cell therapy for B-cell acute lymphoblastic leukemia. JAMA Oncol. 2024;10(6):821–824. 38635232 10.1001/jamaoncol.2024.0473 PMC11190796

[60] Zhou D, Sun Q, Xia J, Gu W, Qian J, Zhuang W, Yan Z, Cheng H, Chen W, Zhu F, et al. Anti-BCMA/GPRC5D bispecific CAR T cells in patients with relapsed or refractory multiple myeloma: A single-arm, single-centre, phase 1 trial. Lancet Haematol. 2024;11(10):e751–e760. 39059405 10.1016/S2352-3026(24)00176-5

[61] Mailankody S, Devlin SM, Landa J, Nath K, Diamonte C, Carstens EJ, Russo D, Auclair R, Fitzgerald L, Cadzin B, et al. GPRC5D-targeted CAR T cells for myeloma. N Engl J Med. 2022;387(13):1196–1206. 36170501 10.1056/NEJMoa2209900 PMC10309537

[62] Li YR, Lyu Z, Chen Y, Fang Y, Yang L. Frontiers in CAR-T cell therapy for autoimmune diseases. Trends Pharmacol Sci. 2024;45(9):839–857. 39147651 10.1016/j.tips.2024.07.005

[63] Chen H, Yu T, Lin L, Xing L, Cho SF, Wen K, Aardalen K, Oka A, Lam J, Daley M, et al. γ-Secretase inhibitors augment efficacy of BCMA-targeting bispecific antibodies against multiple myeloma cells without impairing T-cell activation and differentiation. Blood Cancer J. 2022;12(8):118. 35973981 10.1038/s41408-022-00716-3 PMC9381512

[64] Cowan AJ, Pont MJ, Sather BD, Turtle CJ, Till BG, Libby EN 3rd, Coffey DG, Tuazon SA, Wood B, Gooley T, et al. γ-Secretase inhibitor in combination with BCMA chimeric antigen receptor T-cell immunotherapy for individuals with relapsed or refractory multiple myeloma: A phase 1, first-in-human trial. Lancet Oncol. 2023;24(7):811–822. 37414012 10.1016/S1470-2045(23)00246-2 PMC10783021

[65] Zheng Y, Zhu Q, Li X, Ge T, Wang S, Jia R, Yang L, Wang Y, Zhuang A. Epigenetic reprogramming holds promise in enhancing anti-tumor efficacy of CAR T cell therapy. Biotechnol Adv. 2025;83: Article 108649. 40691879 10.1016/j.biotechadv.2025.108649

[66] Hardman C, Ho S, Shimizu A, Luu-Nguyen Q, Sloane JL, Soliman MSA, Marsden MD, Zack JA, Wender PA. Synthesis and evaluation of designed PKC modulators for enhanced cancer immunotherapy. Nat Commun. 2020;11(1):1879. 32312992 10.1038/s41467-020-15742-7 PMC7170889

[67] Fang C, Xiao G, Wang T, Song L, Peng B, Xu B, Zhang K. Emerging nano-/biotechnology drives oncolytic virus-activated and combined cancer immunotherapy. Research. 2023;6:0108. 37040283 10.34133/research.0108 PMC10079287

[68] Ponterio E, Haas TL, De Maria R. Oncolytic virus and CAR-T cell therapy in solid tumors. Front Immunol. 2024;15:1455163. 39539554 10.3389/fimmu.2024.1455163 PMC11557337

[69] Aboalela MA, Abdelmoneim M, Matsumura S, Eissa IR, Bustos-Villalobos I, Sibal PA, Orikono Y, Takido Y, Naoe Y, Kasuya H. Enhancing mesothelin CAR T cell therapy for pancreatic cancer with an oncolytic herpes virus boosting CAR target antigen expression. Cancer Immunol Immunother. 2025;74(7):202. 40366419 10.1007/s00262-025-04039-7 PMC12078189

[70] Guedan S, Posey AD Jr, Shaw C, Wing A, Da T, Patel PR, McGettigan SE, Casado-Medrano V, Kawalekar OU, Uribe-Herranz M, et al. Enhancing CAR T cell persistence through ICOS and 4-1BB costimulation. JCI Insight. 2018;3(1): Article e96976. 29321369 10.1172/jci.insight.96976 PMC5821198

[71] Herbrich S, Chaib M, Sharma P. ICOS-expressing CAR-T cells mediate durable eradication of triple-negative breast cancer and metastasis. J Immunother Cancer. 2025;13(3): Article e011564. 40107673 10.1136/jitc-2025-011564 PMC11927425

[72] Moreno-Cortes E, Franco-Fuquen P, Garcia-Robledo JE, Forero J, Booth N, Castro JE. ICOS and OX40 tandem co-stimulation enhances CAR T-cell cytotoxicity and promotes T-cell persistence phenotype. Front Oncol. 2023;13:1200914. 37719008 10.3389/fonc.2023.1200914 PMC10502212

[73] Zhang C, Jia J, Heng G, Li Y, Wang M, Chen J, Wang L, Jiang D, Yang Z, Qian C. CD27 agonism coordinates with CD28 and 4-1BB signal to augment the efficacy of CAR-T cells in colorectal tumor. Med Oncol. 2023;40(4):123. 36944898 10.1007/s12032-023-01959-1

[74] Reincke SM, von Wardenburg N, Homeyer MA, Kornau HC, Spagni G, Li LY, Kreye J, Sánchez-Sendín E, Blumenau S, Stappert D, et al. Chimeric autoantibody receptor T cells deplete NMDA receptor-specific B cells. Cell. 2023;186(23):5084–5097.e18. 37918394 10.1016/j.cell.2023.10.001

[75] Liu H, Zhou C, Wang D, Meng H, Zhu S, Zhang J, Mao J, Ye Q. Autoantibodies targeting proteasome subunit alpha type 1 in autoimmune podocytopathies. J Am Soc Nephrol. 2025;36(3):406–419. 39382973 10.1681/ASN.0000000525 PMC11888960

[76] Amatya C, Pegues MA, Lam N, Vanasse D, Geldres C, Choi S, Hewitt SM, Feldman SA, Kochenderfer JN. Development of CAR T cells expressing a suicide gene plus a chimeric antigen receptor targeting signaling lymphocytic-activation molecule F7. Mol Ther. 2021;29(2):702–717. 33129371 10.1016/j.ymthe.2020.10.008 PMC7854354

[77] Wang Z, Li P, Zeng X, Guo J, Zhang C, Fan Z, Wang Z, Zhu P, Chen Z. CAR-T therapy dilemma and innovative design strategies for next generation. Cell Death Dis. 2025;16(1):211. 40148310 10.1038/s41419-025-07454-x PMC11950394

[78] Mestermann K, Giavridis T, Weber J, Rydzek J, Frenz S, Nerreter T, Mades A, Sadelain M, Einsele H, Hudecek M. The tyrosine kinase inhibitor dasatinib acts as a pharmacologic on/off switch for CAR T cells. Sci Transl Med. 2019;11(499): Article eaau5907. 31270272 10.1126/scitranslmed.aau5907 PMC7523030

[79] Fan Y, Duan Y, Chen J, Wang Y, Shang K, Jiang J, Su L, Zhou C, Sadelain M, Huang H, et al. Bispecific killer cell engager-secreting CAR-T cells redirect natural killer specificity to enhance antitumour responses. Nat Biomed Eng. 2025. DOI: 10.1038/s41551-025-01450-4 10.1038/s41551-025-01450-4 40659834

[80] Tang L, Pan S, Wei X, Xu X, Wei Q. Arming CAR-T cells with cytokines and more: Innovations in the fourth-generation CAR-T development. Mol Ther. 2023;31(11):3146–3162. 37803832 10.1016/j.ymthe.2023.09.021 PMC10638038

[81] Hawkins ER, D’Souza RR, Klampatsa A. Armored CAR T-cells: The next chapter in T-cell cancer immunotherapy. Biologics. 2021;15:95–105. 33883875 10.2147/BTT.S291768 PMC8053711

[82] Zhao Y, Chen J, Andreatta M, Feng B, Xie YQ, Wenes M, Wang Y, Gao M, Hu X, Romero P, et al. IL-10-expressing CAR T cells resist dysfunction and mediate durable clearance of solid tumors and metastases. Nat Biotechnol. 2024;42(11):1693–1704. 38168996 10.1038/s41587-023-02060-8

[83] Subklewe M, Magno G, Gebhardt C, Bücklein V, Szelinski F, Arévalo HJR, Hänel G, Dörner T, Zugmaier G, von Bergwelt-Baildon M, et al. Application of blinatumomab, a bispecific anti-CD3/CD19 T-cell engager, in treating severe systemic sclerosis: A case study. Eur J Cancer. 2024;204: Article 114071. 38691878 10.1016/j.ejca.2024.114071

[84] Alexander T, Krönke J, Cheng Q, Keller U, Krönke G. Teclistamab-induced remission in refractory systemic lupus erythematosus. N Engl J Med. 2024;391(9):864–866. 39231352 10.1056/NEJMc2407150

[85] Stockfelt M, Teng YKO, Vital EM. Opportunities and limitations of B cell depletion approaches in SLE. Nat Rev Rheumatol. 2025;21(2):111–126. 39815102 10.1038/s41584-024-01210-9

[86] Zhao J, Lin Q, Song Y, Liu D. Universal CARs, universal T cells, and universal CAR T cells. J Hematol Oncol. 2018;11(1):132. 30482221 10.1186/s13045-018-0677-2 PMC6257951

[87] Zhang L, Wang Y, Xu KL. Mechanisms and prevention strategies of relapse and resistance after BCMA-CAR-T cell in multiple myeloma. Zhonghua Xue Ye Xue Za Zhi. 2021;42(9):778–781. 34753237 10.3760/cma.j.issn.0253-2727.2021.09.014 PMC8607042

[88] Porazzi P, Nason S, Yang Z, Carturan A, Ghilardi G, Guruprasad P, Patel RP, Tan M, Padmanabhan AA, Lemoine J, et al. EZH1/EZH2 inhibition enhances adoptive T cell immunotherapy against multiple cancer models. Cancer Cell. 2025;43(3):537–551.e7. 39983725 10.1016/j.ccell.2025.01.013 PMC13312562

[89] Isshiki Y, Chen X, Teater M, Karagiannidis I, Nam H, Cai W, Meydan C, Xia M, Shen H, Gutierrez J, et al. EZH2 inhibition enhances T cell immunotherapies by inducing lymphoma immunogenicity and improving T cell function. Cancer Cell. 2025;43(1):49–68.e9. 39642889 10.1016/j.ccell.2024.11.006 PMC11732734

[90] Weber EW, Parker KR, Sotillo E, Lynn RC, Anbunathan H, Lattin J, Good Z, Belk JA, Daniel B, Klysz D, et al. Transient rest restores functionality in exhausted CAR-T cells through epigenetic remodeling. Science. 2021;372(6537): Article eaba1786. 33795428 10.1126/science.aba1786 PMC8049103

[91] Wu S, Wei Y, Qiu Y, Ai K, Chen M, Wang H, Zhang H, Cen Q, Liao P, Ding X, et al. Inhibition of VEGF signaling prevents exhaustion and enhances anti-leukemia efficacy of CAR-T cells via Wnt/β-catenin pathway. J Transl Med. 2025;23(1):494. 40307793 10.1186/s12967-024-05907-z PMC12044824

[92] Talbot LJ, Chabot A, Ross AB, Beckett A, Nguyen P, Fleming A, Chockley PJ, Shepphard H, Wang J, Gottschalk S, et al. Redirecting B7-H3.CAR T cells to chemokines expressed in osteosarcoma enhances homing and antitumor activity in preclinical models. Clin Cancer Res. 2024;30(19):4434–4449. 39101835 10.1158/1078-0432.CCR-23-3298 PMC11443211

[93] Mohty R, Gauthier J. Current combinatorial CAR T cell strategies with Bruton tyrosine kinase inhibitors and immune checkpoint inhibitors. Bone Marrow Transplant. 2021;56(11):2630–2636. 34290380 10.1038/s41409-021-01420-9

[94] Zhang Z, Ren C, Xiao R, Ma S, Liu H, Dou Y, Fan Y, Wang S, Zhan P, Gao C, et al. Palmitoylation of TIM-3 promotes immune exhaustion and restrains antitumor immunity. Sci Immunol. 2024;9(101): Article eadp7302. 39546589 10.1126/sciimmunol.adp7302

[95] Gao G, Liao W, Shu P, Ma Q, He X, Zhang B, Qin D, Wang Y. Targeting sphingosine 1-phosphate receptor 3 inhibits T-cell exhaustion and regulates recruitment of proinflammatory macrophages to improve antitumor efficacy of CAR-T cells against solid tumor. J Immunother Cancer. 2023;11(8): Article e006343. 37591632 10.1136/jitc-2022-006343 PMC10441059

[96] Ouyang W, Jin SW, Xu N, Liu WY, Zhao H, Zhang L, Kang L, Tao Y, Liu Y, Wang Y, et al. PD-1 downregulation enhances CAR-T cell antitumor efficiency by preserving a cell memory phenotype and reducing exhaustion. J Immunother Cancer. 2024;12(4): Article e008429. 38589248 10.1136/jitc-2023-008429 PMC11015237

[97] Pozzessere C, Mazini B, Omoumi P, Jreige M, Noirez L, Digklia A, Fasquelle F, Sempoux C, Dromain C. Immune-related adverse events induced by immune checkpoint inhibitors and CAR-T cell therapy: A comprehensive imaging-based review. Cancer. 2024;16(14):2585. 10.3390/cancers16142585 PMC11274393 39061225

[98] Ma X, Shou P, Smith C, Chen Y, Du H, Sun C, Porterfield Kren N, Michaud D, Ahn S, Vincent B, et al. Interleukin-23 engineering improves CAR T cell function in solid tumors. Nat Biotechnol. 2020;38(4):448–459. 32015548 10.1038/s41587-019-0398-2 PMC7466194

[99] Giuffrida L, Sek K, Henderson MA, Lai J, Chen AXY, Meyran D, Todd KL, Petley EV, Mardiana S, Mølck C, et al. CRISPR/Cas9 mediated deletion of the adenosine A2A receptor enhances CAR T cell efficacy. Nat Commun. 2021;12(1):3236. 34050151 10.1038/s41467-021-23331-5 PMC8163771

[100] Lai M, Shao W, Mao J, Ye Q. Revolution in cell therapy: In vivo chimeric-antigen-receptor-T-cell therapy breakthroughs and promises for the future. Research. 2025;8:0917. 41079670 10.34133/research.0917 PMC12509061

[101] Zhang AQ, Hostetler A, Chen LE, Mukkamala V, Abraham W, Padilla LT, Wolff AN, Maiorino L, Backlund CM, Aung A, et al. Universal redirection of CAR T cells against solid tumours via membrane-inserted ligands for the CAR. Nat Biomed Eng. 2023;7(9):1113–1128. 37291434 10.1038/s41551-023-01048-8 PMC10504084

[102] Wang X, Fan R, Mu M, Zhou L, Zou B, Tong A, Guo G. Harnessing nanoengineered CAR-T cell strategies to advance solid tumor immunotherapy. Trends Cell Biol. 2025;35(9):782–798. 39721923 10.1016/j.tcb.2024.11.010

[103] Mi J, Ye Q, Min Y. Advances in nanotechnology development to overcome current roadblocks in CAR-T therapy for solid tumors. Front Immunol. 2022;13: Article 849759. 35401561 10.3389/fimmu.2022.849759 PMC8983935

[104] Wang Y, Jin S, Zhuang Q, Liu N, Chen R, Adam SA, Jin J, Sun J. Chimeric antigen receptor natural killer cells: A promising antitumor immunotherapy. MedComm. 2023;4(6): Article e422. 38045827 10.1002/mco2.422 PMC10691297

[105] Imai C, Iwamoto S, Campana D. Genetic modification of primary natural killer cells overcomes inhibitory signals and induces specific killing of leukemic cells. Blood. 2005;106(1):376–383. 15755898 10.1182/blood-2004-12-4797 PMC1895123

[106] Xiao L, Cen D, Gan H, Sun Y, Huang N, Xiong H, Jin Q, Su L, Liu X, Wang K, et al. Adoptive transfer of NKG2D CAR mRNA-engineered natural killer cells in colorectal cancer patients. Mol Ther. 2019;27(6):1114–1125. 30962163 10.1016/j.ymthe.2019.03.011 PMC6554529

[107] Töpfer K, Cartellieri M, Michen S, Wiedemuth R, Müller N, Lindemann D, Bachmann M, Füssel M, Schackert G, Temme A. DAP12-based activating chimeric antigen receptor for NK cell tumor immunotherapy. J Immunol. 2015;194(7):3201–3212. 25740942 10.4049/jimmunol.1400330

[108] Xie G, Dong H, Liang Y, Ham JD, Rizwan R, Chen J. CAR-NK cells: A promising cellular immunotherapy for cancer. EBioMedicine. 2020;59: Article 102975. 32853984 10.1016/j.ebiom.2020.102975 PMC7452675

[109] Rafei H, Basar R, Acharya S, Hsu YS, Liu P, Zhang D, Bohn T, Liang Q, Mohanty V, Upadhyay R, et al. CREM is a regulatory checkpoint of CAR and IL-15 signalling in NK cells. Nature. 2025;643(8073):1076–1086. 40468083 10.1038/s41586-025-09087-8 PMC12286855

[110] Boulifa A, Franzén AS, Raftery MJ, Radecke C, Pecher G. Chimeric antigen receptor (CAR)-NK92 cells effective against glioblastoma, breast- and pancreatic cancer in vitro and in a murine xenograft model of ovarian cancer. Cancer Cell Int. 2025;25(1):260. 40646591 10.1186/s12935-025-03865-0 PMC12255008

[111] Marin D, Li Y, Basar R, Rafei H, Daher M, Dou J, Mohanty V, Dede M, Nieto Y, Uprety N, et al. Safety, efficacy and determinants of response of allogeneic CD19-specific CAR-NK cells in CD19+ B cell tumors: A phase 1/2 trial. Nat Med. 2024;30(3):772–784. 38238616 10.1038/s41591-023-02785-8 PMC10957466

[112] Chen Q, Xia M, Tang M, Liu M, Yan D, Chen W, Xu S, Xu Z, Chen YH, Zhang G, et al. DR5 CAR-NK cells demonstrate superior scalability and potent antitumor activity with favorable safety. Mol Ther. 2025;33(12):6063–6081. 41103033 10.1016/j.ymthe.2025.10.035 PMC12703157

[113] Heim C, Hartig L, Weinelt N, Moser LM, Salzmann-Manrique E, Merker M, Wels WS, Tonn T, Bader P, Klusmann JH, et al. Bortezomib promotes the TRAIL-mediated killing of resistant rhabdomyosarcoma by ErbB2/Her2-targeted CAR-NK-92 cells via DR5 upregulation. Mol Ther Oncol. 2024;32(2): Article 200802. 38706988 10.1016/j.omton.2024.200802 PMC11067460

[114] Fantini M, Arlen PM, Tsang KY. Potentiation of natural killer cells to overcome cancer resistance to NK cell-based therapy and to enhance antibody-based immunotherapy. Front Immunol. 2023;14:1275904. 38077389 10.3389/fimmu.2023.1275904 PMC10704476

[115] Lei W, Liu H, Deng W, Chen W, Liang Y, Gao W, Yuan X, Guo S, Li P, Wang J, et al. Safety and feasibility of 4-1BB co-stimulated CD19-specific CAR-NK cell therapy in refractory/relapsed large B cell lymphoma: A phase 1 trial. Nat Cancer. 2025;6(5):786–800. 40251398 10.1038/s43018-025-00940-3 PMC12122374

[116] Li W, Wang X, Zhang X, Aziz AUR, Wang D. CAR-NK cell therapy: A transformative approach to overcoming oncological challenges. Biomolecules. 2024;14(8):1035. 39199421 10.3390/biom14081035 PMC11352442

[117] Peng L, Sferruzza G, Yang L, Zhou L, Chen S. CAR-T and CAR-NK as cellular cancer immunotherapy for solid tumors. Cell Mol Immunol. 2024;21(10):1089–1108. 39134804 10.1038/s41423-024-01207-0 PMC11442786

[118] Ward MB, Jones AB, Krenciute G. Therapeutic advantage of combinatorial chimeric antigen receptor T cell and chemotherapies. Pharmacol Rev. 2025;77(1): Article 100011. 39952691 10.1124/pharmrev.124.001070 PMC13095475

[119] Sinelshchikov D, Belmonte-Beitia J, Italia M. A mathematical model of CAR-T cell therapy in combination with chemotherapy for malignant gliomas. Chaos. 2025;35(6): Article 063104. 40455204 10.1063/5.0260252

[120] Liu W, Liu W, Zou H, Chen L, Huang W, Lv R, Xu Y, Liu H, Shi Y, Wang K, et al. Combinational therapy of CAR T-cell and HDT/ASCT demonstrates impressive clinical efficacy and improved CAR T-cell behavior in relapsed/refractory large B-cell lymphoma. J Immunother Cancer. 2024;12(4): Article e008857. 38631712 10.1136/jitc-2024-008857 PMC11029269

[121] Ma X, Zhang W, Zeng M, Asavasupreechar T, Kang S, Li Y, Yu L. Systemic tumor regression with synergy therapy: Radiotherapy and CAR-T. Cell Death Discov. 2024;10(1):479. 39578426 10.1038/s41420-024-02245-3 PMC11584735

[122] Szlasa W, Sztuder A, Kaczmar-Dybko A, Maciejczyk A, Dybko J. Efficient combination of radiotherapy and CAR-T—A systematic review. Biomed Pharmacother. 2024;174: Article 116532. 38574625 10.1016/j.biopha.2024.116532

[123] Fan J, Adams A, Sieg N, Heger JM, Gödel P, Kutsch N, Kaul D, Teichert M, von Tresckow B, Bücklein V, et al. Potential synergy between radiotherapy and CAR T-cells—A multicentric analysis of the role of radiotherapy in the combination of CAR T cell therapy. Radiother Oncol. 2023;183: Article 109580. 36842663 10.1016/j.radonc.2023.109580

[124] Schett G, Müller F, Taubmann J, Mackensen A, Wang W, Furie RA, Gold R, Haghikia A, Merkel PA, Caricchio R, et al. Advancements and challenges in CAR T cell therapy in autoimmune diseases. Nat Rev Rheumatol. 2024;20(9):531–544. 39107407 10.1038/s41584-024-01139-z

[125] Li YR, Lyu Z, Shen X, Fang Y, Yang L. Boosting CAR-T cell therapy through vaccine synergy. Trends Pharmacol Sci. 2025;46(2):180–199. 39755457 10.1016/j.tips.2024.12.004

[126] Hamieh M, Mansilla-Soto J, Rivière I, Sadelain M. Programming CAR T cell tumor recognition: Tuned antigen sensing and logic gating. Cancer Discov. 2023;13(4):829–843. 36961206 10.1158/2159-8290.CD-23-0101 PMC10068450

[127] Agliardi G, Dias J, Rampotas A, Garcia J, Roddie C. Accelerating and optimising CAR T-cell manufacture to deliver better patient products. Lancet Haematol. 2025;12(1):e57–e67. 39510106 10.1016/S2352-3026(24)00273-4

[128] Xu J, Wang BY, Yu SH, Chen SJ, Yang SS, Liu R, Chen LJ, Hou J, Chen Z, Zhao WH, et al. Long-term remission and survival in patients with relapsed or refractory multiple myeloma after treatment with LCAR-B38M CAR T cells: 5-year follow-up of the LEGEND-2 trial. J Hematol Oncol. 2024;17(1):23. 38659046 10.1186/s13045-024-01530-z PMC11040812

[129] Jurgens EM, Firestone RS, Chaudhari J, Hosszu K, Devlin SM, Shah UA, Landa J, McAvoy DP, Lesokhin AM, Korde N, et al. Phase I trial of MCARH109, a G protein-coupled receptor class C group 5 member D (GPRC5D)-targeted chimeric antigen receptor T-cell therapy for multiple myeloma: An updated analysis. J Clin Oncol. 2025;43(5):498–504. 39631041 10.1200/JCO-24-01785 PMC11798713

[130] Schultz LM, Jeyakumar N, Kramer AM, Sahaf B, Srinagesh H, Shiraz P, Agarwal N, Hamilton M, Erickson C, Jacobs A, et al. CD22 CAR T cells demonstrate high response rates and safety in pediatric and adult B-ALL: Phase 1b results. Leukemia. 2024;38(5):963–968. 38491306 10.1038/s41375-024-02220-y PMC12740236

[131] Yin J, Cui QY, Dai HP, Qu CJ, Li Z, Kang LQ, Cui W, Tian XP, Zhu XM, Yu L, et al. CD19 CAR-T in relapsed t(8;21) AML: A single-center prospective phase II clinical trial. J Hematol Oncol. 2025;18(1):53. 40329358 10.1186/s13045-025-01708-z PMC12057151

[132] Ghobadi A, Bachanova V, Patel K, Park JH, Flinn I, Riedell PA, Bachier C, Diefenbach CS, Wong C, Bickers C, et al. Induced pluripotent stem-cell-derived CD19-directed chimeric antigen receptor natural killer cells in B-cell lymphoma: A phase 1, first-in-human trial. Lancet. 2025;405(10473):127–136. 39798981 10.1016/S0140-6736(24)02462-0 PMC11827677

